# Ectoine Production from Biogas in Waste Treatment Facilities: A Techno-Economic and Sensitivity Analysis

**DOI:** 10.1021/acssuschemeng.1c06772

**Published:** 2021-12-15

**Authors:** Víctor Pérez, Jose Luis Moltó, Raquel Lebrero, Raúl Muñoz

**Affiliations:** † Institute of Sustainable Processes, University of Valladolid, Dr. Mergelina, s/n, 47011 Valladolid, Spain; ‡ Department of Chemical Engineering and Environmental Technology, School of Industrial Engineering, University of Valladolid, Dr. Mergelina, s/n, 47011 Valladolid, Spain; § Activatec Ltd, Biocity, Pennyfoot St, NG11GFNottingham, United Kingdom

**Keywords:** Biogas valorization, Biorefinery, Ectoine, Haloalkaliphilic methanotrophic bacteria, Techno-economic assessment, Sensitivity analysis

## Abstract

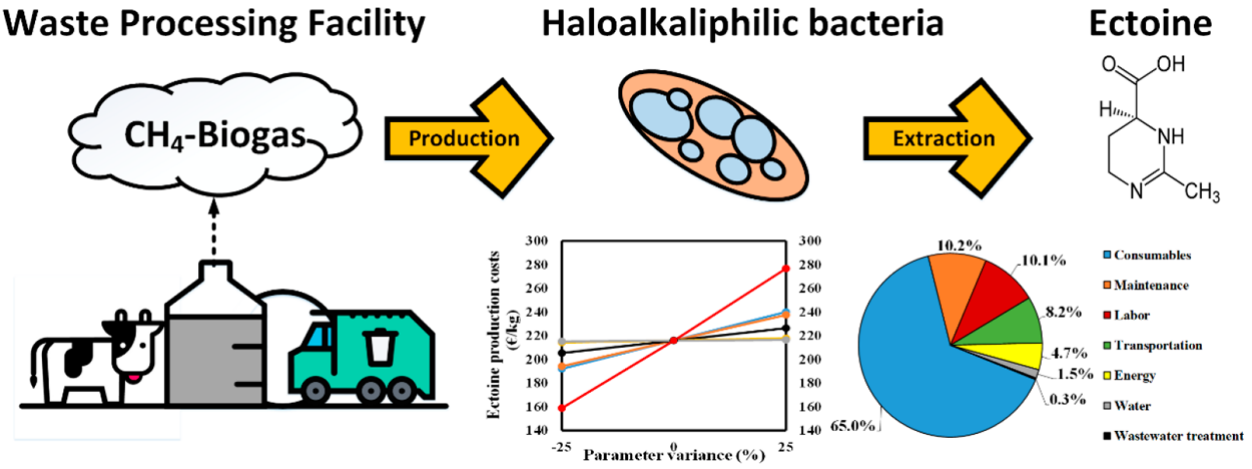

The capacity of haloalkaliphilic methanotrophic bacteria to synthesize ectoine from CH_4_-biogas represents an opportunity for waste treatment plants to improve their economic revenues and align their processes to the incoming circular economy directives. A techno-economic and sensitivity analysis for the bioconversion of biogas into 10 t ectoine·y^–1^ was conducted in two stages: (I) bioconversion of CH_4_ into ectoine in a bubble column bioreactor and (II) ectoine purification via ion exchange chromatography. The techno-economic analysis showed high investment (4.2 M€) and operational costs (1.4 M€·y^–1^). However, the high margin between the ectoine market value (600–1000 €·kg^–1^) and the estimated ectoine production costs (214 €·kg^–1^) resulted in a high profitability for the process, with a net present value evaluated at 20 years (NPV_20_) of 33.6 M€. The cost sensitivity analysis conducted revealed a great influence of equipment and consumable costs on the ectoine production costs. In contrast to alternative biogas valorization into heat and electricity or into low added-value bioproducts, biogas bioconversion into ectoine exhibited high robustness toward changes in energy, water, transportation, and labor costs. The worst- and best-case scenarios evaluated showed ectoine break-even prices ranging from 158 to 275 €·kg^–1^, ∼3–6 times lower than the current industrial ectoine market value.

## Abstract 2

A techno-economic and sensitivity assessment of biogas valorization into ectoine, the highest added-value bioproduct produced with methanotrophic bacteria.

## Introduction

In the past decade, the construction of biogas plants associated with the treatment of wastewater, agro-industrial residues, and urban waste in Europe has grown exponentially from 6 227 in 2011 to 18 943 operative biogas plants in 2019.^1^ The main motivation behind this growth has been the production of renewable electricity from biogas, which has increased concomitantly from 66 TWh in 2011 to 167 TWh in 2019 in Europe.^1^ Nevertheless, in the past few years, the high competition in the European renewable energy market combined with the rapid drop in production costs of competing renewable energies (−82% and −39% drop between 2010 and 2019 for solar and wind energies, respectively) and the elevated capital (400–1100 €·kW^–1^) and operational costs (0.01–0.02 €·kWh^–1^) of electricity and heat cogeneration (CHP) systems have stalled the growth of this biogas valorization alternative, with a marginal increase of 4.3% in the period 2015–2019.^1−4^ In this regard, a recent techno-economic analysis has demonstrated the excessive dependence of biogas-to-energy facilities on the extension of fiscal incentives.^5^ However, fiscal exemptions such as feed in tariffs or carbon credits are no longer available for renewable energy production as policy-makers and governments have recently focused their attention on the transformation of current waste treatment plants into circular biorefineries, able to produce marketable products from waste streams.

In this context, alternative biogas valorization pathways such as the production of biomethane (renewable natural gas) and platform chemicals such as methanol, polyhydroxyalkanoates (PHA), or single cell protein from biogas components (mainly methane (CH_4_) and carbon dioxide (CO_2_)) have rapidly developed and attracted significant research efforts from academia and industry.^6,7^ The transition from the current linear anaerobic digestion plants to next-generation circular waste biorefineries might help in adapting the processes to the increasingly restrictive environmental policies, in line with the European Green New Deal and Circular Economy Directives, while boosting the economic feasibility of waste treatment plants by reducing the influence of the fluctuating energy market on the final economic balance of the plant.^8,9^ The best example of this changing trend is the increasing number of public–private initiatives aimed at demonstrating at semi-industrial scale the technical, economic, and environmental feasibility of waste biorefineries. Particularly, different European consortia have included in their biorefinery concepts the biological transformation of biogas into: biomethane (INCOVER and URBIOFIN), PHA (URBIOFIN), biostimulants (CIRCULAR BIOCARBON) or ectoine (DEEP PURPLE). These demo-scale projects, together with an extensive investigation work at laboratory scale, have consistently evidenced the high economic and environmental potential of these technologies at industrial scale.^7,9,10^

Today, CH_4_-based bioproducts struggle to compete in price against their oil-based or sugar-based counterparts mainly due to the high energy demand required for CH_4_ and oxygen (O_2_) gas–liquid mass transfer and the low productivity of methanotrophic fermentation processes.^11,12^ These technological barriers are especially relevant for the production of low added-value products such as PHA (4–20 €·kg^–1^), single cell protein (0.5–1 €·kg^–1^), or methanol (0.5–2 €·kg^–1^), whose operational costs often exceed their market selling prices, thus hindering their production in waste treatment facilities.^8^

However, the recent discovery of the capacity of haloalkaliphilic methanotrophic bacteria to accumulate high amounts of ectoine (up to 230 mg ectoine·g biomass^–1^), a bacterial osmotic protector with a high industrial interest in the cosmetic industry, has opened the door to the production of high added-value products from biogas.^13,14^ Ectoine, with a market price ranging from 600 to 1000 €·kg^–1^ and an annual demand in the range of 20 t, has been traditionally produced via sugar-based fermentation with *Halomonas elongate* in a process called “bio-milking”.^15,16^ This biotechnological process presents high production costs due to the use of high quality carbon sources (e.g., glucose) and sterile conditions, and an expensive downstream processing.^17,18^ Therefore, the use of CH_4_-biogas as a widely available and low-cost substrate might contribute to the reduction of ectoine production costs and can be regarded as an opportunity for waste management companies to invest in circular economy concepts. However, the techno-economic feasibility of upscaling the production process and the influence of commodity prices and capital costs on biogas-based ectoine production remain unknown.

In this study, a techno-economic evaluation of ectoine production from biogas was conducted, with special attention to the production of ectoine in a bubble column bioreactors (BCB) and to the ectoine purification process via ion exchange chromatography (IEX). In addition, a sensitivity analysis was performed to assess the influence of labor and transportation costs, commodity prices, capital costs, and the interest and tax rates on the final market price of ectoine.

To the best of the authors knowledge, this study constitutes the first comprehensive techno-economic and sensitivity analysis focusing on the production of high added-value products from biogas.

## Materials and Methods

All calculations were performed in Excel Sheets and have been corroborated with global and elemental mass balances. Mass and energy balances were performed assuming an ideal gas behavior given the low pressure and temperatures of the streams. Mass and energy balances as well as stream tables can be consulted in the Supporting Information (SI Figures S1–S3; Tables S1–S7). A detailed compilation of the equipment design calculation has been included in the SI. The text includes all the relevant parameters for a reliable reproduction of the process design, economic calculations, and sensitivity analysis herein performed.

### Process Design

**Figure 1:**
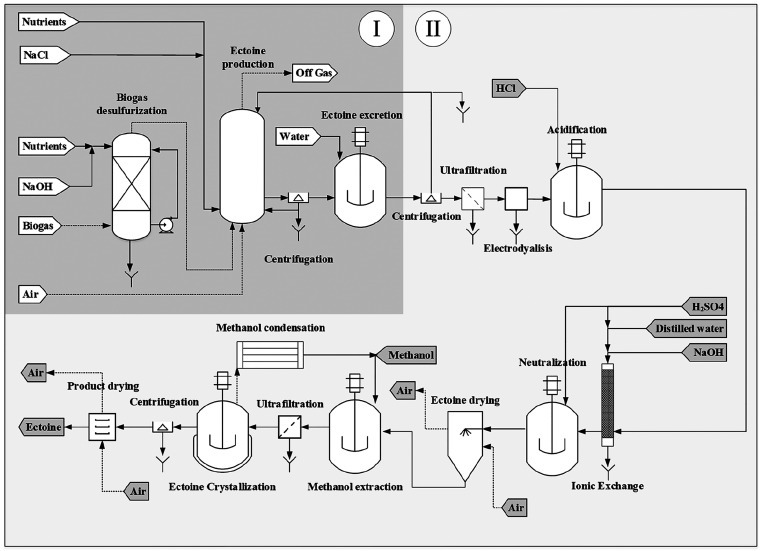
Simplified process flow diagram for CH_4_-biogas bioconversion into ectoine. The process was divided into two different stages: (I) ectoine biosynthesis from biogas and (II) ectoine extraction and purification.

A waste treatment plant with a continuous biogas production of 1000 N m^3^·h^–1^ was selected as a model centralized anaerobic digestion plant constructed in medium and large municipalities. In this type of plant, 40% of the total biogas production is typically used for internal energy provision via CHP and therefore, the remaining 600 N m^3^·h^–1^ are available to be further valorized. In this study, 67 N m^3^·h^–1^ of the remaining biogas stream was considered as a carbon source for the production of 10 t·y^–1^ ectoine. The process was divided into two different stages: (I) ectoine biosynthesis from biogas and (II) ectoine extraction and purification (Figure 1). A more detailed process flow diagram, including all the auxiliary equipment, is included in the SI (Figures S1–S3).

### Ectoine Biosynthesis from Biogas

Prior to biogas valorization into ectoine, a biogas desulfurization stage was designed for preventing corrosion in downstream piping and equipment. Biological anoxic desulfurization was selected as the model technology given its low operational costs, reduced environmental impact, and high H_2_S removal efficiency (H_2_S-RE).^19^ In this process, sulfur-oxidizing bacteria use nitrate (NO_3_^–^) instead of oxygen as the electron acceptor for the oxidation of H_2_S into SO_4_^2–^.^20^ A 4 m^3^ (height, *H* = 3.1 m and diameter, *D*) = 1.3 m) biotrickling filter packed with a mixture of activated carbon and inert material was designed with an empty bed residence time (EBRT) of 3 min and an H_2_S-RE of 99%. A molar nitrogen-to-sulfur ratio of 2.5 was guaranteed by continuously spraying the packing media with a sodium nitrate (NaNO_3_) and micronutrients solution at a trickling liquid velocity of 10 m·h^–1^.^21^ A constant pH of 7 was maintained via addition of 5 M sodium hydroxide solution (NaOH).

A mixed culture of haloalkaliphilic methanotrophic bacteria was selected for the production of ectoine from CH_4_-biogas. The use of mixed methanotrophic cultures has been demonstrated to be an efficient method supporting long-term operation and process robustness, given its inherent prevention of culture contamination.^22^ Despite the fact that the bacterial population structure might be highly variable depending on the inoculum and the culture and environmental conditions, previous work at lab-scale under nonsterile conditions has shown a predominance of ectoine producing methanotrophs such as *Methylomicrobium buryatense* and *Methylomicrobium japanense* species in these mixed cultures.^23^ A NaCl concentration of 6%w·w^–1^ has been reported in the literature as the optimal salinity for the accumulation of ectoine in haloalkaliphilic methanotrophic cultures.^23,24^ The bioreactor was operated under continuous mode at a dilution rate of 0.4 d^–1^. A mineral medium solution containing 41.0 g NaNO_3_·L^–1^, 82.5 g NaCl·L^–1^, and trace concentrations of micronutrients was continuously added to support haloalkaliphilic methanotrophic bacteria growth and ectoine synthesis. Recent studies have shown that high copper concentrations promote ectoine excretion from the cell in haloalkaliphilic bacteria.^24^ In this study, ectoine excretion under high salinity conditions was considered negligible, given the trace levels of copper in the mineral medium. A tungsten concentration of 0.07 mg·L^–1^ was supplemented to the liquid medium in order to prevent the formation of formic acid, which typically exhibits a significant inhibitory effect on the elimination capacity of methane (CH_4_-EC).^25^ A specific biomass production yield of 0.4 g biomass · g CH_4_^–1^ and an ectoine accumulation of 70 mg ectoine· g biomass^–1^ were chosen according to previous results at laboratory scale.^23^ The stoichiometric formulas of biomass and ectoine were C_4_H_8_O_2_N and C_6_H_10_N_2_O_2_, respectively. A mineralization ratio of 0.7 mol CO_2_·mol CH_4_^–1^ and an oxygen demand of 1.5 mol O_2_·mol CH_4_^–1^ were used according to eqs 1–3:
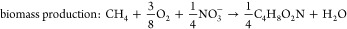
1
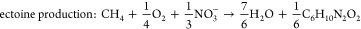
2
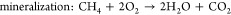
3

A BCB with a total volume of 196 m^3^ (*H* = 30 m; *D* = 2.9 m) and a gas EBRT of 1.2 h was calculated as the model bioreactor to support an effective gas–liquid mass transfer of CH_4_ (biogas) and O_2_ (air). The calculation process has been detailed in the SI. Perfect mixing in the BCB was assumed given the high turbulence induced by the high biogas/air gas flow and the high height-to-diameter ratio (*H*/*D*) (10) of the bioreactor, resulting in a CH_4_ removal efficiency (CH_4_-RE) of 90%. CH_4_-RE was defined as the percentage of methane being eliminated in the bioreactor by the action of methanotrophic bacteria according to eq 4, where *Q* stands for the volumetric gas flow in the inlet and outlet streams, and *Y*_CH4_ stands for the molar fraction of CH_4_ in the gas phase in the inlet and outlet gas streams.
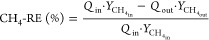
4

The calculated outlet gas composition was 1.4/17.1/79.3/2.1% for CH_4_/CO_2_/N_2_/O_2_, respectively. Therefore, further valorization of the outlet gas stream was not considered as an energy vector given the high amount of inert compounds (N_2_ and CO_2_) and the concentration of CH_4_ and O_2_ below the explosion limits (CH_4_ 5–15% and O_2_ > 13%). Additionally, recycling the outlet stream into the bioreactor would result in a decrease in the gas–liquid gradient and a high energy cost for the recompression of the gas stream.

Previous studies on biogas valorization in BCBs have shown that CH_4_-EC constitutes the main biotechnological limitation given its large influence on the capital investment costs (TIC).^7^ CH_4_-EC has been defined as the CH_4_ mass flow eliminated by volumetric unit of bioreactor according to eq 5, where *Q* stands for the inlet and outlet volumetric flow of gas streams, *Y*_CH4_ refers to the molar fraction of CH_4_ in the inlet and outlet streams, and *V* represents the liquid volume in the bioreactor.
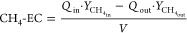
5

However, to date there is not available information in the literature concerning CH_4_-EC in large-scale BCBs. For the purpose of this study, a CH_4_-EC value of 148 g CH_4_ m^–3^ h^–1^ was extrapolated from commercial BCBs treating 30%v·v^–1^ CO streams, which can reach up to 1 kg CO·m^–3^·h^–1^.^26^ A CO volumetric mass transfer coefficient (kla_CO_) was calculated with eq 6, where CO-EC stands for the CO elimination capacity, *C*_COin_ represents the inlet CO gas concentration, *H*_CO_ refers to the dimensionless Henry’s law constant of CO (29.81 at 288.15 K and 3.78 atm), and *C*_LCO_ for the bulk aqueous CO concentration, considered negligible under mass transfer limitation scenarios.^27^
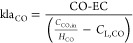
6

The volumetric CH_4_ mass transfer coefficient (CH_4_-kla) was calculated according to eq 7, where *V*_m_ stands for the molar volume at the normal boiling point of CH_4_ (35.05 m^3^·kg^–1^) and CO (32.74 m^3^·kg^–1^).^28^
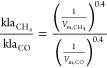
7

CH_4_-EC was calculated according to eq 8, where kla_i_ stands for the volumetric mass transfer coefficient of substance i, *C*_CH4,in_ stands for the inlet CH_4_ gas concentration, *H*_CH4_ for the dimensionless Henry’s law constant of CH_4_ (43.03 at 288.15 K and 3.78 atm), and *C*_L,CH4_ for the bulk aqueous CH_4_ concentration, considered negligible under mass-transfer limitation scenarios.^27^
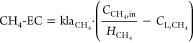
8

### Ectoine Extraction and Purification

Haloalkaliphilic methanotrophic bacteria containing ectoine was harvested and centrifuged to reach a biomass concentration of 200 g biomass·L^–1^. An aliquot of the liquid fraction of 10% was daily wasted from the system to avoid the accumulation of secondary metabolites. The concentrated biomass stream was subjected to a hyposmotic shock in a nonsaline medium to promote the excretion of 85% of the total intracellular ectoine.^29,30^ The process was carried out in a 104.1 L (*H* = 0.6 m; *D* = 0.5 m) continuous stirred tank reactor (CSTR) with a hydraulic retention time (HRT) of 5 min.^26^ Biomass containing only 15% of the initial intracellular ectoine was subsequently centrifuged to a concentration of 200 g biomass·L^–1^ and recirculated to the bioreactor. A fraction of the biomass was continuously wasted to maintain an average biomass residence time of 9 d, preventing biomass activity decay, similarly to the industrial biomilking process with *Halomonas Elongate*.^11^ The aqueous solution containing the extracted ectoine was desalinized via subsequent ultrafiltration and electrodialysis. A 58.2 m^2^ ultrafiltration membrane system was designed with a typical permeate flux of 15 L·m^–2^·h^–1^, a pressure drop of 300 mbar, and a biomass recovery of 99%. Accordingly, a typical permeate flux of 45 L·m^–2^·h^–1^, a pressure drop of 200 mbar, and a concentration factor of 25 were selected for desalination with a 17.5 m^2^ electrodialysis system.

A two-step IEX and methanol crystallization process has been identified as the most efficient and scalable method for ectoine concentration and purification, capable of providing a high recovery and purity of the product.^18,31^ This process was simulated in this paper with a product recovery and purity of 62% and 97%, respectively. Prior to the isolation of ectoine via IEX, the liquid stream containing ectoine was acidified to pH 2 by addition of 10 M HCl in a 1.7 m^3^ (*H* = 1.5 m; *D* = 1.2 m) CSTR operated at a HRT of 1 h.^18^ Then, the ectoine broth was pumped into a column packed with an ion-exchange resin and selectively adsorbed. A resin bed volume (BV) of 500 L (*H* = 4 m; *D* = 0.4 m) was needed to achieve an ectoine recovery of 90%. A high performance ion-exchange resin (DOWEX 50w × 8) with an adsorbing capacity of 0.1 kg ectoine·kg resin^–1^ and a density of 800 kg·m^3^ was selected.^32^ The adsorbed ectoine was washed with 2 BV of 98%w·w^–1^ H_2_SO_4_ and 2 BV of distilled water to remove impurities. Finally, the ectoine was eluted with 6 BV of 1.3 M NaOH, of which 4 BV were discarded. Subsequently, the liquid was neutralized to pH 7 via addition of 98%w·w^–1^ H_2_SO_4_ in a 40.4 L (*H* = 0.4; *D* = 0.3) CSTR with an EBRT of 1 h.

In a later step, the product was dried to a moisture content of 5%w·w^–1^ in a 400 L spray dryer operated at 0.3 atm and heated via low pressure steam (2 bar). The spray drying system was designed with a specific evaporation rate of 100 kg water·m^–3^·h^–1^. Prior to ectoine crystallization, the solid product was dissolved into methanol (10 kg methanol·kg ectoine^–1^) in a 16.4 L (*H* = 0.3 m; *D* = 0.3 m) CSTR operated at an HRT of 1 h. An additional ultrafiltration (1.1 m^2^) step was included before ectoine crystallization to remove insoluble matter, especially the Na_2_SO_4_ produced during the previous neutralization step. Ectoine crystallization was performed in a 12.3 L (*H* = 0.3; *D* = 0.3 m) CSTR operated at a HRT of 1 h. 99% of the methanol was evaporated at 65 °C with the use of low pressure steam (2 bar) and subsequently recovered in a 3.6 m^2^ condenser using cooling water as the refrigerant (15 °C). The ectoine crystallized was then centrifuged to remove the remaining methanol. The final product was obtained after a second drying step in a 0.9 m^2^ tray dryer with warm air (20 °C).

### Economic Analysis

The economic analysis was performed using as indicators the net present value evaluated at 20 years (NPV_20_), the payback period (PP), and the internal rate of return (IRR). The NPV_20_ was calculated from the free cash flow (FCF) according to eq 9:
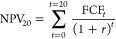
9where *t* represents the financial period in years, and *r* stands for the interest rate (5%). For the calculation of FCF, the TIC were attributed to year 0 and a circulating capital over the TIC of 5% was included in year 1. A linear depreciation of 20 years and a tax rate of 30% were selected for the calculation. A median selling price of 600 €·kg ectoine^–1^ was used to estimate the NPV_20_. The IRR was calculated as the value of *r* that makes NPV_20_ = 0. Finally, the PP was estimated as the first period in which the accumulated FCF is positive. The break-even price was used for estimating the ectoine production costs as the value of sales that guarantees a NPV_20_ equal to zero.

### Capital Costs

TIC were calculated according to Lang’s Method. This method is based on a series of factors for the estimation of the TIC from the sum of the individual prices of equipment (PEC). A Lang factor of 4.09 has been calculated as optimum for solid–liquid processes like the one evaluated in this paper (Table 1).^33^ Equipment prices were obtained from Matches’ database, a commonly used quotation tool that compiles order-of-magnitude estimations for more than 275 types of equipment.^34^ Matches’ equipment prices were updated to 2020-€ considering a dollar exchange rate of 1.09 €·$^–1^ and an accumulated dollar inflation rate of 1.1 in the period 2014–2020. Prices from equipment not included in Matches’ database were obtained from quotations of national and international companies and literature review (Table S6).

**Table 1:** Influence of Labor Cost on the Lang Factor Calculation

	base case	–25%	25%
**equipment**	1.00	1.00	1.00
+ equipment installation labor^a^	0.38	0.29	0.48
+ instrumentation and controls	0.12	0.12	0.12
+ piping	0.31	0.31	0.31
+ electrical installations	0.10	0.10	0.10
+ buildings	0.29	0.29	0.29
+ yard improvements^a^	0.10	0.08	0.13
+ service facilities	0.54	0.54	0.54
+ land	0.06	0.06	0.06
**direct plant cost**	2.90	2.78	3.02
+ engineering and supervision^a^	0.32	0.24	0.40
+ construction expenses^a^	0.34	0.26	0.43
**direct and indirect costs**	3.56	3.28	3.85
+ contractor’s fee	0.18	0.16	0.19
+ contingency	0.36	0.33	0.38
total depreciable costs**(Lang Factor)**	4.09	3.77	4.42

a Wage dependent parameters.

### Operational Costs

Operational costs were calculated as the sum of consumables (raw materials, chemical reagents, and utilities), transportation cost of raw materials and products, maintenance costs, labor costs, and wastewater treatment cost. Given the high geographical variability in commodity prices, Madrid (Spain) was selected as the model city for the estimation, presenting a worldwide average cost for energy and water selling prices (Table 2). Consumables and commodities requirements (energy, water, steam, cooling water, reagents, and raw materials) were calculated according to mass and energy balances (Tables S1–S7; Figures S1–S3). Typical values for energy requirements in centrifuges (1 kWh·m^–3^), mixers (0.2 kW·m^–3^), and electrodialysis (7 kWh·m^–3^) were selected according to the literature.^35−37^ Energy requirements for pumps were calculated according to eq 10, where *P*_pump_ represents the power in kW, *Q* stands for the volumetric flow expressed in m^3^·s^–1^, Δ*P* is the pressure drop in kPa, and 0.7 is the electrical efficiency of pumps and compressors.
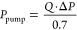
10

**Table 2:** Summary of Utility and Commodity Prices Used in Madrid as Model Country

consumable	price	unit
energy	0.10	€·kWh^–1^
water	1.89	€·m^–3^
steam	0.14	€·kg^–1^
cooling water	0.00006	€·kg^–1^
distilled water	0.07	€·kg^–1^
methanol	2.00	€·kg^–1^
H_2_SO_4_ 98%w·w^–1^	0.20	€·kg^–1^
NaOH	0.46	€·kg^–1^
ion exchange resin^a^	342.57	€·kg^–1^
HCl 32%w·w^–1^	0.25	€·kg^–1^
packing media	1.50	€·kg^–1^
NaCl	0.07	€·kg^–1^
NaNO_3_	0.64	€·kg^–1^
micronutrients	0.19	€·kg^–1^

a Including treatment cost as hazardous waste.

Energy requirements for blowers and compressors were calculated according to eqs 11 and 12, where *P*_blower_ represents the power in kW, *P*_is_ stands for the isentropic power in kW, 0.7 is the electrical blower efficiency, γ is the adiabatic coefficient, *T*_o__u__t_ refers to the gas isentropic outlet temperature, *T*_i__n_ represents the gas inlet temperature, *P*_m_ is the gas molecular weight, and *Q* stands for the inlet volumetric flow.
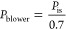
11
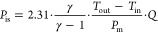
12

Transportation costs for raw materials and ectoine products were considered comparable to other petrochemical products (60 €·t^–1^). The cost of ectoine transportation was equally distributed between production and extraction costs. Yearly maintenance costs of 3.5% over the TIC were selected as recommended by industrial waste operators. To the best of the authors’ knowledge, there is not a standard method for evaluating the labor cost of biogas upgrading processes integrated in larger facilities. Therefore, labor costs were evaluated assuming a total of 192 person-h·week^–1^, as recommended by industrial waste operators. Two full-time operators with 8 h-shift during week days (2 person · 1 shift · 8 h·shift^–1^ · 5 d·week^–1^ = 80 person-h·week^–1^) and 2 part-time operators during the evening and night shifts during the whole week (2 person · 2 shift^–1^ · 4 h·shift^–1^ · 7 d·week^–1^ = 112 person-h·week^–1^) were herein considered. A wage of 14.5 €·person-h^–1^ was considered as average salary in Madrid (Spain).^38^ Wastewater treatment costs were considered comparable to domestic wastewater (0.2 €·m^–3^) given the low organic load of these waste streams.

### Sensitivity Analysis

A sensitivity analysis for the most relevant consumables and commodities was performed to validate the results of the techno-economic analysis and provide a reliable error margin. In this context, the selling prices of water and energy and the costs of reagents, labor, and transportation were increased and decreased by 25%. The Lang Factor was calculated in each scenario assuming that some of the factors are wage-dependent (Table 2). PEC was also increased and decreased by 25% to assess the influence of the assumptions made in the equipment cost estimation. The worst- and best-case scenarios were calculated considering a 25% decrease/increase in all the items at once. Additionally, a sensitivity analysis was performed on the interest rate (5%, 10%, and 20%) and the tax rate (30%, 40%, and 50%) to evaluate the robustness of the economic analysis.

## Results and Discussion

### Capital Costs

**Figure 2:**
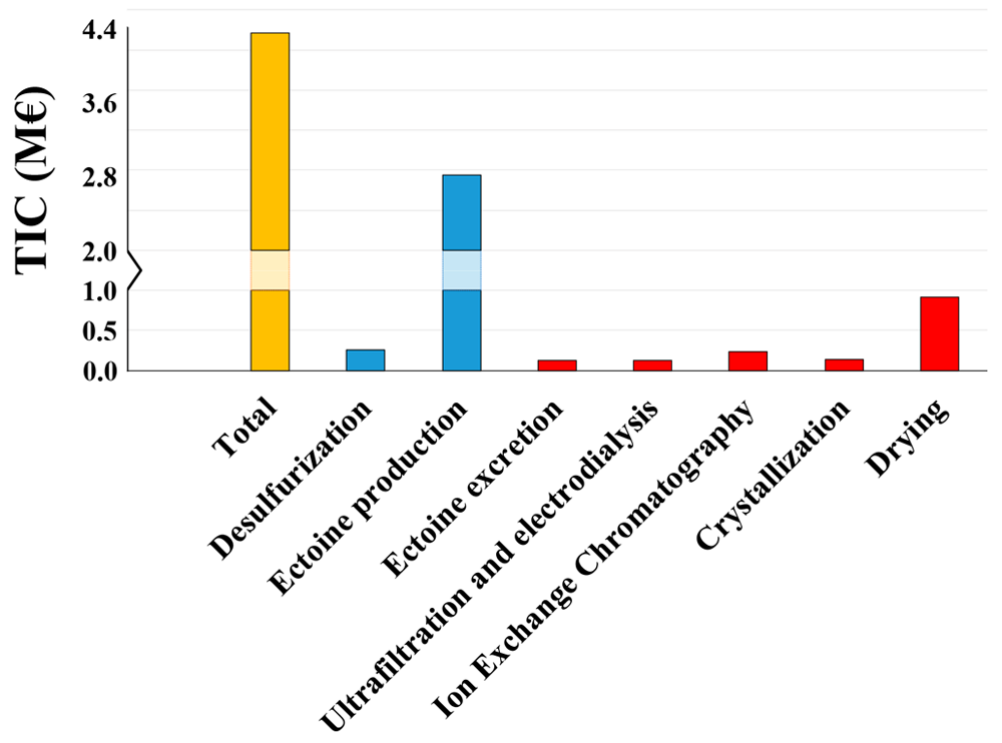
Total Investment Costs (TIC) of the biogas valorization into ectoine process. In yellow bars, the TIC of the process. In blue bars, the TIC of the different items for the ectoine production from biogas. In red bars, the TIC of the different items for the downstream processing of ectoine.

The total PEC for ectoine production from biogas accounted for 1.03 M€, with 0.66 M€ and 0.37 M€ corresponding to the bioconversion of CH_4_-biogas into ectoine and to the ectoine extraction and purification, respectively. Surprisingly, the PEC of ectoine production represented 63.8% of the total PEC, while 36.2% was allocated to the downstream processing. The high PEC differences between ectoine production and downstream stages can be explained by the volumetric flow rates of gas (67 N m^3^ biogas·h^–1^ and 280 N m^3^ air·h^–1^) and liquid (6 m^3^ water·h^–1^) streams processed at similar HRT, which incurred a great variability in equipment size. The application of the Lang’s Method resulted in a TIC of 4.21 M€, with 2.69 M€ and 1.52 M€ for the ectoine production and downstream processing, respectively (Figure 2).

The commissioning of the BCB, with an individual volume of 195 m^3^, represented the main equipment cost with 0.6 M€. The purchase of this bioreactor accounted for 56.8% of the total PEC. The elevated price of the BCB (3000 €·m^–3^) was due to the need for high-quality materials that resist the high corrosion induced by the high salinity of the mineral medium and the additional safety measures required for the ATEX application derived from mixing biogas and air.^39^ These results are in good agreement with previous techno-economic analyses studying the bioconversion of CH_4_ into PHA, which identified BCBs as the most expensive pieces of equipment.^5,40^ The biotrickling filter dedicated to biogas desulfurization constituted the second most expensive equipment in the biogas bioconversion process with 53 048 €.

The equipment showing the highest PEC in the downstream processing stage were the drying units with 0.22 M€, accounting for 59.6% of the downstream PEC and 21.6% of the overall process PEC. Ectoine excretion, ultrafiltration, and electrodialysis, IEX, and crystallization represented 8.0%, 8.3%, 8.9%, and 15.2% over the total downstream PEC, respectively.

### Operational Costs

**Figure 3:**
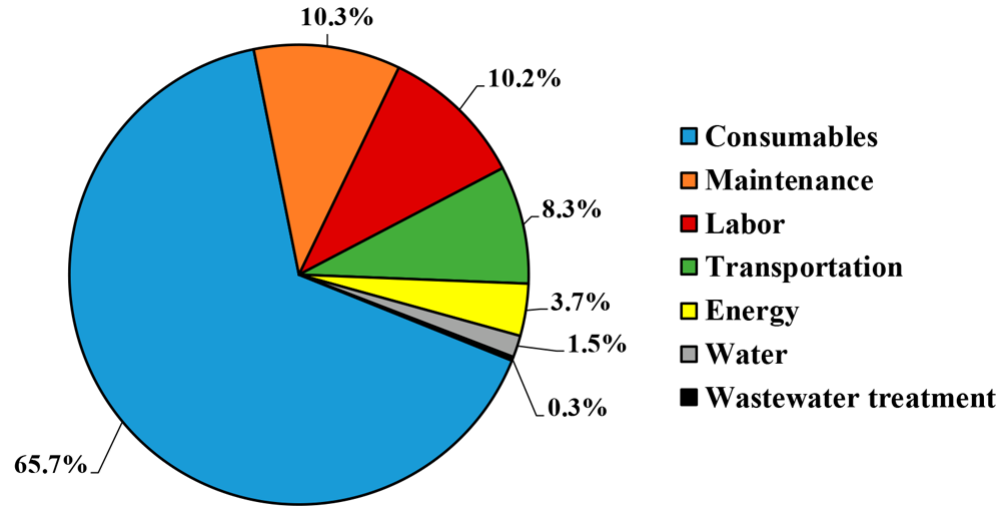
Individual share of the operational costs for ectoine production from biogas.

The operational costs for biogas-based ectoine production accounted for 1.4 M€·y^–1^, with 0.3 M€·y^–1^ and 1.1 M€·y^–1^ associated with ectoine production and purification, respectively. The purchase of consumables (chemicals, raw materials, and utilities) was the most significant operational cost (0.9 M€·y^–1^), accounting for 65.7% of the total operational costs (Figure 3). The ion exchange resins for ectoine adsorption represented the highest raw material cost with 0.6 M€·y^–1^, due to the use of expensive high performance resins (343 €·kg^–1^) and to their limited lifespan (80 d). In this context, the bulk purchase of this raw material (1825 kg·y^–1^) could help in reducing the effective selling price of ectoine. The ectoine retention performance was used as resin selection criteria in this study, while the maximum NPV_20_ might be achieved using resins with a lower performance but a longer lifespan.

Maintenance and labor costs represented the second and third largest operational cost, with 10.3% and 10.2% of the total cost share (Figure 3). Maintenance costs were herein calculated as 3.5% of TIC, and therefore the high PEC of the installation and in particular the elevated yearly maintenance of the BCB, can explain the high contribution of maintenance costs.

In addition, transportation costs were identified as a non-negligible operational cost, with 8.3% of the total operational costs (Figure 3). This can be explained by the high amount of mineral salts required for the growth of haloalkaliphilic methanotrophic bacteria (199.4 t·y^–1^ and 121.0 t·y^–1^ of NaCl and NaNO_3_, respectively). However, this cost was marginal compared to those reported by Shazad and co-workers (2013) during the production of PHA from slaughter waste, which accounted for almost 50% of the total production costs.^41^ The transportation cost of products represented only 2.5% of the total transportation costs given the reduced amount of ectoine produced (10.0 t·y^–1^). These results suggest that the use of in situ produced biogas as carbon source for bacterial fermentations entails a significant reduction in the bioproducts production costs. Notwithstanding, the influence of transportation costs on the final ectoine price should be taken into account in the design of next-generation biorefineries as it might be of relevance if the distance to mineral salts suppliers and potential ectoine buyers was significantly increased.

Finally, the costs of energy and water represented only 3.7% and 1.5% of the total operational costs, respectively. The relevance of these commodities might change significantly depending on the location of the plant and should not be neglected when studying the viability of future ectoine production plants. Wastewater treatment costs were negligible compared to the total operational costs, representing a share of only 0.3%.

### Economic Analysis

As a result of the process, 10 t·y^–1^ of ectoine were obtained from 67 N m^3^ h^–1^ of biogas, which was in line with the estimated global ectoine demand ranging 10–20 t·y^–1^. The calculations resulted in an overall ectoine productivity of 17 mg ectoine produced·m^–3^ biogas treated. A break-even price for ectoine, calculated as the selling price at which NPV_20_ becomes 0, of 214 €·kg^–1^ was estimated. This value represented a 3-fold decrease against the lowest reported market values for ectoine production with *Halomonas Elongate* (600–1000 €·kg^–1^). Interestingly, 66.6% of the break-even price was allocated to the operational costs, while the remaining 33.4% was attributed to the TIC and its amortization.

In view of the wide difference between the calculated ectoine production costs (214 €·kg^–1^) and the median ectoine market value (600 €·kg^–1^), an outstandingly positive NPV_20_ of 33.6 M€ was obtained. Accordingly, a IRR of 70.4% and a PP of 1.5 year (2 years) were obtained. The high profitability of CH_4_-to-ectoine process has been previously reported by Cantera and co-workers in a preliminary techno-economic study for the coproduction of PHA, single cell protein, extracellular polysaccharides, and ectoine from CH_4_ diluted streams.^36^ In this regard, given the wide difference between production cost and current ectoine selling price, and the limited number of companies dedicated to the production of ectoine, it is worth questioning if ectoine is currently sold following a cost-based or a market-based strategy. If ectoine commercialization follows a cost-based strategy, then the production of ectoine from biogas using methanotrophic bacteria could have the potential to displace the current industrial routes with *Halomonas Elongate*. On the contrary, if ectoine is following a market-based strategy, the selling prices could drop in the future. In any scenario, the low production costs herein presented (3–6 times lower than the current selling price of ectoine) guarantee a current and future economic feasibility of the biogas-to-ectoine process.

In this context, the results indicated a high profitability of the valorization of biogas into ectoine regardless of the 20–30% level of estimation of techno-economic analysis like the one presented in this paper. Regardless of the low worldwide demand of ectoine, in the range of 20 t·y^–1^, which could incur in a limited impact of ectoine production from biogas at a global scale, these results demonstrate that bioproduct production with methanotrophic bacteria should not be restricted to bulk and low added-value products such as PHA, SCP, or methanol but also to fine chemicals such as ectoine. The results herein presented open the door to a great opportunity for waste management companies and biogas producers in general to invest in the production of high added-value products from biogas.

However, CH_4_ and O_2_ gas–liquid mass transfer, biomass concentration, and bacterial bioproduct productivity in aerated bioreactors have been identified as key factors determining the economic feasibility of bioprocesses.^42^ Thus, the influence of the economy of scale and the aforementioned biotechnological limitations on the final ectoine production costs should be evaluated in the future.

### Sensitivity Analysis

**Figure 4:**
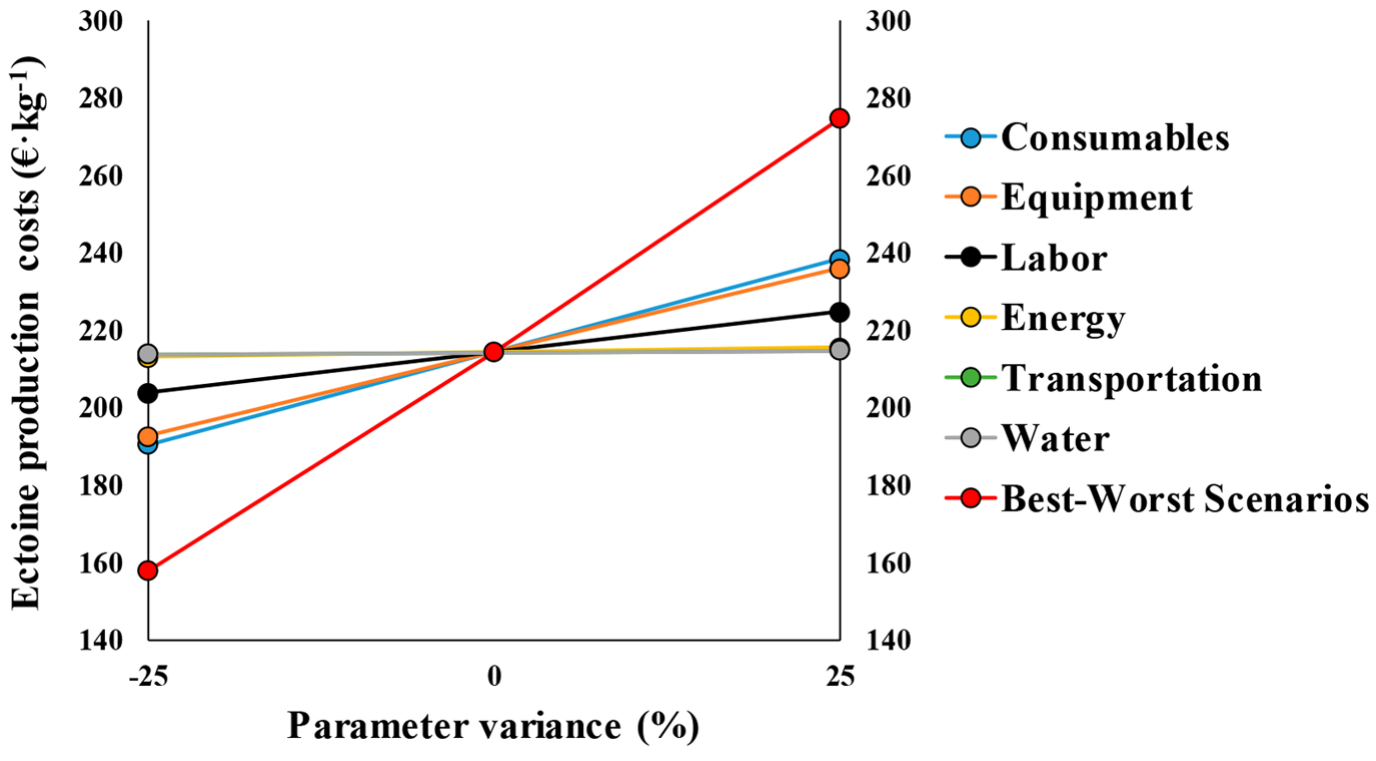
Sensitivity analysis of ectoine production costs toward the most relevant capital and operational costs. All the parameters were increased and decreased individually by 25%.

A sensitivity analysis was herein performed to assess the impact of the assumptions made during the calculation of the operational and capital costs. In this context, the highest sensitivity of ectoine production costs was observed toward changes in the purchase price of consumables (chemicals, raw materials, and utilities). A 25% increase/decrease in the consumables purchase costs resulted in ectoine production costs ranging from 191 to 238 €·kg^–1^ (Figure 4).

The ectoine production costs also showed a high sensitivity toward the PEC estimation. A ±25% variance in the PEC resulted in a ±10.1% change in the production costs of ectoine (Figure 4). Interestingly, even if PEC increased by 1 order of magnitude (from 1.03 M€ to 10.3 M€), the biogas-based ectoine would be still market-competitive with a break-even price of 991.7 €·kg^–1^.

The sensitivity analysis showed a mild influence of labor costs on the final product price, with ectoine production costs varying by 4.9% with a 25% change on the average wage. At this point it should be stressed that changes in labor costs increased operational costs by raising the average wage of plant operators from 10.9 €·h^–1^ to 18.1 €·h^–1^, but also entailed a change in the Lang Factor from 3.77 to 4.42, thus affecting the TIC (Table 2). Despite the change in TIC from 4.2 M€ to 4.5 M€, the ectoine production cost calculated was 225 €·kg^–1^.

Variations in energy and water purchase prices resulted in negligible changes in the ectoine production costs of 0.6 and 0.3%, respectively. These results implied that ectoine production cost in waste treatment facilities might be profitable regardless of the variability of commodity prices. In contrast, recent techno-economic analyses indicated that the production of low added-value products such as biopolymers from biogas exhibited an inherently high influence of these commodities on the economic feasibility of the technology.^5^ Despite transportation costs represented a significant share of the total operational costs (8.3%), a 25% change on the unitary transportation costs (from 60 €·t^–1^ to 45 and 75 €·t^–1^) induced negligible changes on the ectoine production costs from 213.8 to 214.8 €·kg^–1^. Interestingly, increasing transportation unitary costs by 1 order of magnitude (from 60 to 600 €·t^–1^) increased ectoine production costs to only 232.5 €·kg^–1^. These results showed a high robustness of the process toward changes on the most influential parameters for other low added-value bioproducts and expand the economic viability of ectoine production in waste treatment facilities to all sort of socio-economic contexts in terms of commodities (water and energy) costs. The different cost sensitivity of low added- and high added-value bioproducts produced by methanotrophs in waste treatment plants enriches the current dissertation on the scientific community on the roadmap for future and successful CH_4_-biorefineries.

The worst and best case scenarios for ectoine production from biogas were calculated assuming a 25% increase/decrease in all the aforementioned parameters. The evaluation of NPV_20_ and IRR in the worst- and best-case scenarios showed a high variability ranging from 28.4 to 38.6 M€ and from 46.8 to 112.3%, respectively. The calculated PP varied slightly with the 25% increase/decrease between 0.96 (1 year) and 2.35 (3 years). Finally, ectoine production costs ranging from 158 to 275 €·kg^–1^ were estimated in the best and worst case scenarios.

**Figure 5:**
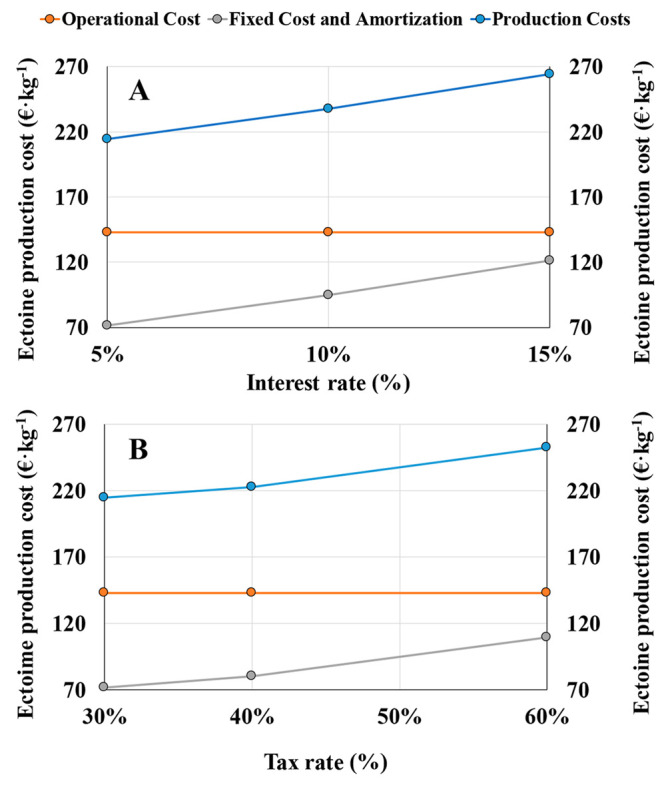
Sensitivity analysis of ectoine production costs toward changes in (A) interest rate, (B) tax rate.

Variations in the interest rate (*r*) from 5% to 10% and 15% resulted in ectoine production costs of 214, 238, and 264 €·kg^–1^, respectively. This increase in the ectoine production costs was correlated to the increase in the fixed and amortization costs of ectoine from 72 €·kg^–1^ to 95 €·kg^–1^ and 121 €·kg^–1^ for *r* values of 5%, 10%, and 15%, respectively (Figure 5A). The operational costs were not affected by the variation of *r*, with a constant value of 143 €·kg^–1^. Besides the increase of the fixed and amortization costs, the high margin between ectoine production costs and selling price, resulted in very positive NPV_20_ values in all the scenarios studied, with 33.6 M€ at *r* = 5%, 21.6 M€ at *r* = 10%, and 14.7 M€ at *r* = 15%. The PP observed in all the scenarios studied remained below 2 years, with 1.5, 1.6, and 1.8 years for *r* values of 5%, 10% and 15%, respectively.

Similarly, an increase in the tax rate from 30% to 40% and 60% resulted in an increase of the ectoine production costs from 214 €·kg^–1^ to 223 €·kg^–1^ and 252 €·kg^–1^, respectively (Figure 5B). In this case, a fixed cost and amortization of 72, 80, and 110 €·kg^–1^ and NPV_20_ values of 33.6M€, 28 M€, and 17.3 M€ were calculated for tax rates of 30%, 40%, and 60%, respectively. Besides the changes in the ectoine production costs and the decrease of NPV_20_, high IRR of 70%, 60%, and 40% were obtained for tax rates of 30%, 40%, and 60%, respectively. In addition, the PP calculated remained below 3 years in all the scenarios studied, with 1.5, 1.8, and 2.8, for tax rates of 30%, 40%, and 60%, respectively. These results point to an outstanding process economic robustness of the biogas bioconversion into ectoine herein studied.

To the best of the authors’ knowledge, these results represent the lowest reported value for ectoine production at large scale and constitute a proof-of-concept of the key role of biogas as a low-cost substrate in the future of next-generation biorefineries.

## Conclusions

This work constituted the first techno-economic study of the large-scale production of ectoine from biogas in waste treatment plants. The results indicated a high profitability of the process with a payback time below 3 years in all the scenarios evaluated. Ectoine break-even prices in the best and worst case scenarios considered entailed a 3- to 6-fold decrease in the ectoine production costs when compared to the current production via long-time fermentation with *Halomonas elongate*, mainly due to the use of CH_4_-biogas as a low-cost carbon substrate for the growth of haloalkaliphilic bacteria. The process showed a high sensitivity toward the purchase cost of equipment and consumables (chemical reagents, raw materials, and utilities). On the contrary, the sensitivity analysis revealed a high robustness toward changes on water and energy prices, labor, and transportation costs. In summary, this study demonstrated that large-scale production of high added-value products from biogas represents a highly profitable alternative to the current utilization of biogas as energy source, but also a much more feasible valorization pathway than the production of low added-value bioproducts. However, the influence of certain techno-economic aspects such as the economy of scale or the microbial bioconversion yields of methane into ectoine on the development of future cost-effective biogas biorefineries must be further investigated.

## Supporting Information Available

Figures S1–S3, Detailed process flow diagram of the biogas bioconversion into ectoine; Table S1, stream table of the biogas bioconversion into ectoine process; Table S2, global mass balance of the ectoine biosynthesis from biogas; Table S3, global mass balance of the ectoine extraction and purification; Table S4, elemental mass balance of the ectoine biosynthesis from biogas; Table S5, elemental mass balance of the ectoine extraction and purification; Table S6, summary of the purchased equipment cost and total investment cost; Table S7, summary of the individual equipment energy and power consumption; and a list of the equations used in the design and dimensioning of the equipment (PDF)

## Abbreviations

BCB: bubble column bioreactor
BV: bed volume
CHP: combined heat and power
D: equipment diameter
EBRT: empty bed residence time
EC: elimination capacity
FCF: free cash flow
*H*: equipment height
HRT: hydraulic retention time
IEX: ion exchange chromatography
IRR: internal rate of return
NPV_**20**_: net present value
PEC: purchased equipment cost
PHA: polyhydroxyalkanoate
PP: payback period
RE: removal efficiency
TIC: total investment costs
