# Supplementary material for: Ectoine Production from Biogas in Waste Treatment Facilities: A Techno-Economic and Sensitivity Analysis
Source: ACS Sustain Chem Eng. 2021 Dec 15;9(51):17371–80. doi: 10.1021/acssuschemeng.1c06772 (PMC8715504; doi:10.1021/acssuschemeng.1c06772)
Supplement: Supplementary file 1 — sc1c06772_si_001.pdf [file sc1c06772_si_001.pdf]

SUPPORTING INFORMATION

Ectoine production from biogas in waste treatment facilities: a techno-economic and sensitivity analysis

Víctor Pérez [a,b], Jose Luis Moltó [c], Raquel Lebrero [a,b] and Raúl Muñoz [a,b]*

[a]Institute of Sustainable Processes, University of Valladolid, Dr. Mergelina, s/n, 47011, Valladolid (Spain).

[b]Department of Chemical Engineering and Environmental Technology, School of Industrial Engineering, University of Valladolid, Dr. Mergelina, s/n, 47011, Valladolid (Spain).

[c]Activatec Ltd, Biocity, Pennyfoot St, NG11GF, Nottingham (United Kingdom).

*Corresponding e-mail: mutora@iq.uva

Number of pages: 31

Number of tables: 7

Number of figures: 3

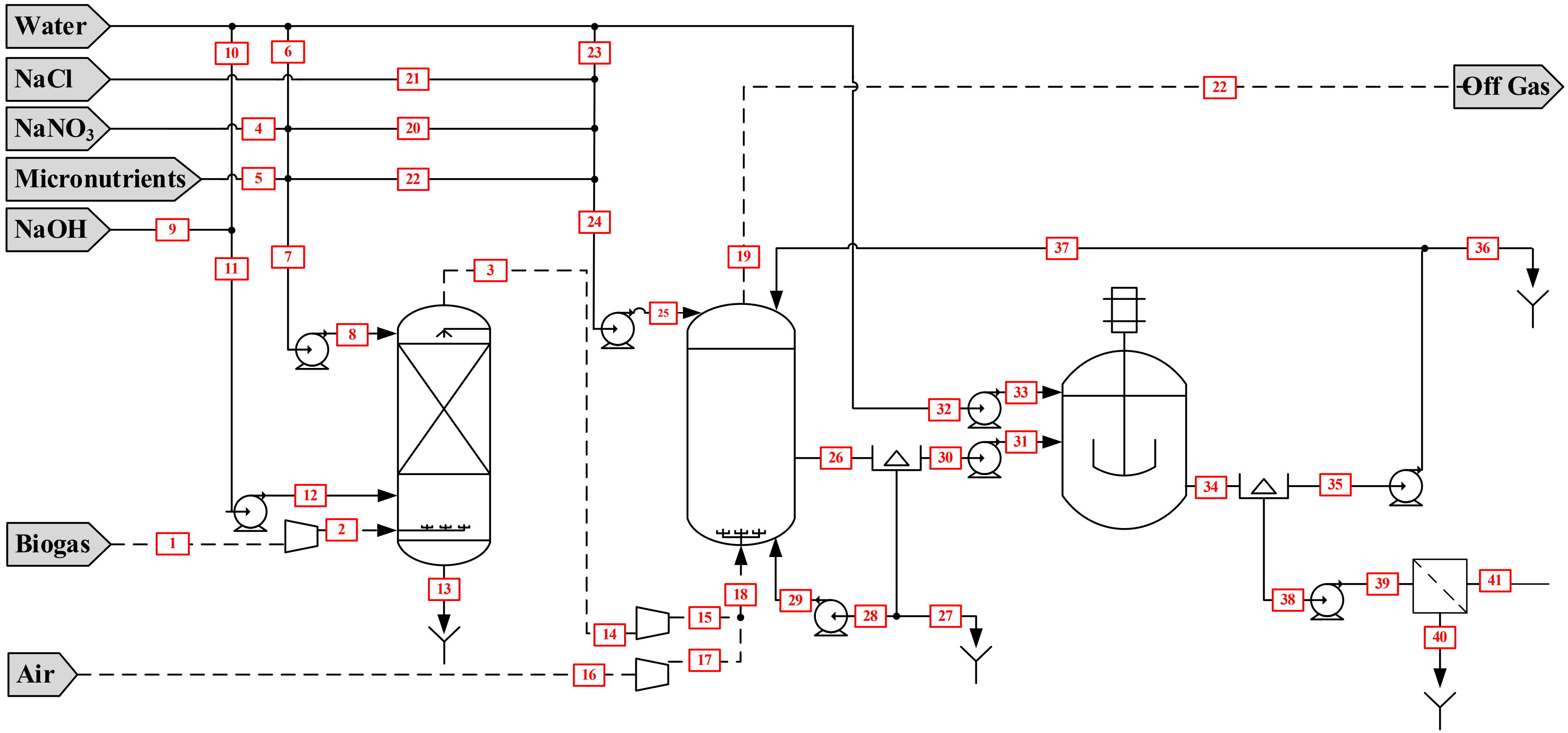

Figure S1. Process flow diagram of the biogas bioconversion into ectoine. Stream numbering is detailed with red labels. (Part 1/3).

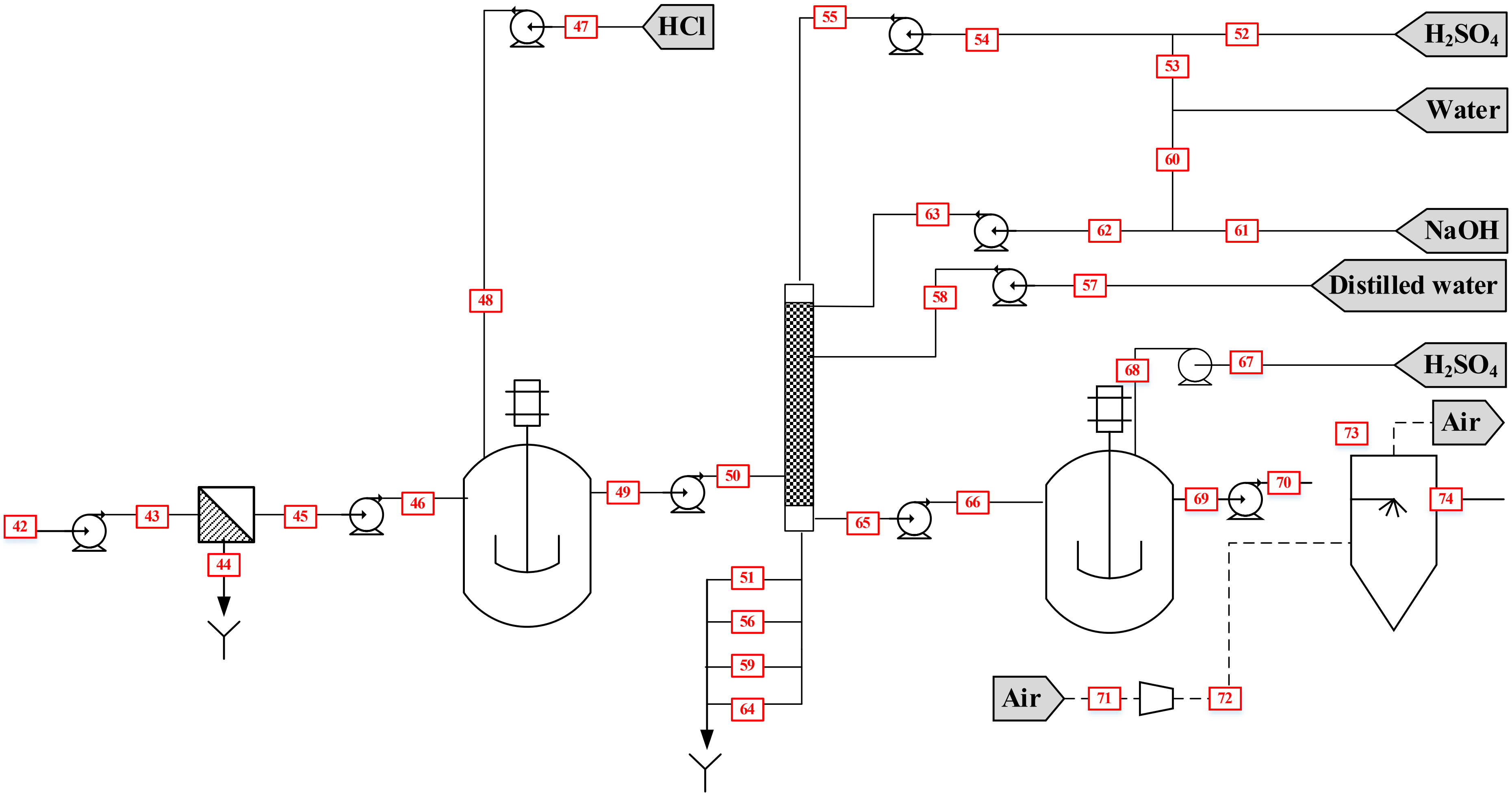

Figure S2. Process flow diagram of the biogas bioconversion into ectoine. Stream numbering is detailed with red labels. (Part 2/3).

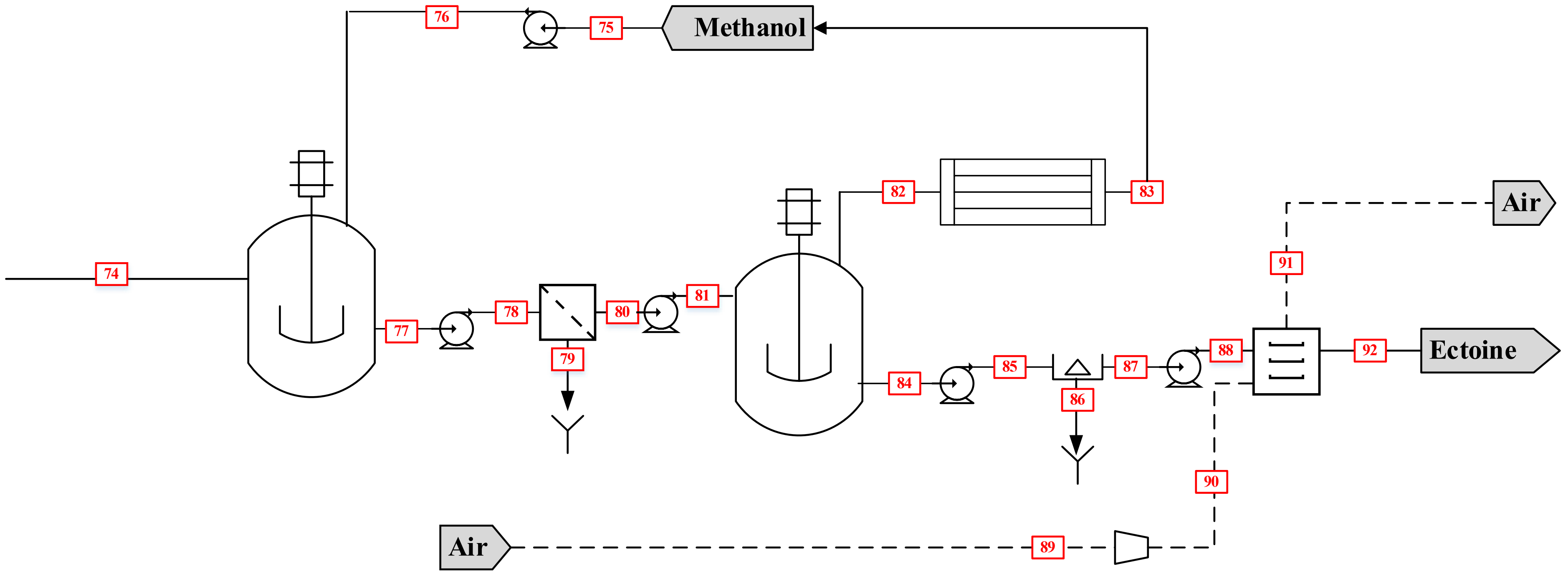

Figure S3. Process flow diagram of the biogas bioconversion into ectoine. Stream numbering is detailed with red labels. (Part 3/3).

Table S1. Mass balance of the production of ectoine from biogas. In columns, the different streams according to Figures S1-S3. (Part 1/8).

**Stream**	**1**	**2**	**3**	**4**	**5**	**6**	**7**	**8**	**9**	**10**	**11**	**12**	**13**
**P (atm)**	1.00	1.17	1.00	1.00	1.00	1.00	1.00	1.10	1.00	1.00	1.00	1.10	1.00
**T (K)**	288.15	299.06	288.15	288.15	288.15	288.15	288.15	288.15	288.15	288.15	288.15	288.15	288.15
**Mass flow (kg·d^{-1})**	1928.92	1928.92	1941.65	60.34	0.53	1206.90	1267.77	1267.77	11.30	56.52	67.83	67.83	1322.86
**CH_4 (kg·d^{-1})**	690.31	690.31	690.29	0.00	0.00	0.00	0.00	0.00	0.00	0.00	0.00	0.00	0.02
**CO_2 (kg·d^{-1})**	1104.66	1104.66	1098.38	0.00	0.00	0.00	0.00	0.00	0.00	0.00	0.00	0.00	0.00
**N_2 (kg·d^{-1})**	50.22	50.22	59.66	0.00	0.00	0.00	0.00	0.00	0.00	0.00	0.00	0.00	0.00
**O_2 (kg·d^{-1})**	11.47	11.47	30.76	0.00	0.00	0.00	0.00	0.00	0.00	0.00	0.00	0.00	0.00
**H_2S (kg·d^{-1})**	9.78	9.78	0.10	0.00	0.00	0.00	0.00	0.00	0.00	0.00	0.00	0.00	0.00
**$NaNO_3$ (kg·d^{-1})**	0.00	0.00	0.00	60.34	0.00	0.00	0.00	0.00	0.00	0.00	0.00	0.00	0.00
**NaCl (kg·d^{-1})**	0.00	0.00	0.00	0.00	0.00	0.00	0.00	0.00	0.00	0.00	0.00	0.00	0.00
**Methanol (kg·d^{-1})**	0.00	0.00	0.00	0.00	0.00	0.00	0.00	0.00	0.00	0.00	0.00	0.00	0.00
**Na (kg·d^{-1})**	0.00	0.00	0.00	0.00	0.00	0.00	16.32	16.32	0.00	0.00	6.50	6.50	22.82
**Cl (kg·d^{-1})**	0.00	0.00	0.00	0.00	0.00	0.00	0.00	0.00	0.00	0.00	0.00	0.00	0.00
**NO_3- (kg·d^{-1})**	0.00	0.00	0.00	0.00	0.00	0.00	44.02	44.02	0.00	0.00	0.00	0.00	0.06
**NaOH (kg·d^{-1})**	0.00	0.00	0.00	0.00	0.00	0.00	0.00	0.00	11.30	0.00	0.00	0.00	0.00
**OH^- (kg·d^{-1})**	0.00	0.00	0.00	0.00	0.00	0.00	0.00	0.00	0.00	0.00	4.81	4.81	0.00
**H^+ (kg·d^{-1})**	0.00	0.00	0.00	0.00	0.00	0.00	0.00	0.00	0.00	0.00	0.00	0.00	0.00
**SO_4^{-2} (kg·d^{-1})**	0.00	0.00	0.00	0.00	0.00	0.00	0.00	0.00	0.00	0.00	0.00	0.00	27.28
**HCl (kg·d^{-1})**	0.00	0.00	0.00	0.00	0.00	0.00	0.00	0.00	0.00	0.00	0.00	0.00	0.00
**H_2SO_4 (kg·d^{-1})**	0.00	0.00	0.00	0.00	0.00	0.00	0.00	0.00	0.00	0.00	0.00	0.00	0.00
**Micronutrients (kg·d^{-1})**	0.00	0.00	0.00	0.00	0.53	0.00	0.53	0.53	0.00	0.00	0.00	0.00	0.53
**Water (kg·d^{-1})**	0.00	0.00	0.00	0.00	0.00	1206.90	1206.90	1206.90	0.00	56.52	56.52	56.52	1268.51
**Biomass (kg·d^{-1})**	0.00	0.00	0.00	0.00	0.00	0.00	0.00	0.00	0.00	0.00	0.00	0.00	3.64
**Ectoine (kg·d^{-1})**	0.00	0.00	0.00	0.00	0.00	0.00	0.00	0.00	0.00	0.00	0.00	0.00	0.00
**Na_2SO_4 (kg·d^{-1})**	0.00	0.00	0.00	0.00	0.00	0.00	0.00	0.00	0.00	0.00	0.00	0.00	0.00
**Others (kg·d^{-1})**	62.48	62.48	62.48	0.00	0.00	0.00	0.00	0.00	0.00	0.00	0.00	0.00	0.00

Table S1. Mass balance of the production of ectoine from biogas. In columns, the different streams according to Figures S1-S3. (Part 2/8).

**Stream**	**14**	**15**	**16**	**17**	**18**	**19**	**20**	**21**	**22**	**23**	**24**	**25**
**P (atm)**	1.00	3.78	1.00	3.78	3.78	1.10	1.10	1.00	1.00	1.00	1.00	1.00
**T (K)**	288.15	391.71	288.15	422.28	416.40	288.15	288.15	288.15	288.15	288.15	288.15	288.15
**Mass flow (kg·d^{-1})**	1941.65	1941.65	8777.00	8777.00	10718.65	9226.97	9226.97	271.18	546.17	2.89	6621.14	7441.37
**CH_4 (kg·d^{-1})**	690.29	690.29	0.00	0.00	690.29	69.03	69.03	0.00	0.00	0.00	0.00	0.00
**CO_2 (kg·d^{-1})**	1098.38	1098.38	4.19	4.19	1102.57	2270.51	2270.51	0.00	0.00	0.00	0.00	0.00
**N_2 (kg·d^{-1})**	59.66	59.66	6621.27	6621.27	6680.93	6680.91	6680.91	0.00	0.00	0.00	0.00	0.00
**O_2 (kg·d^{-1})**	30.76	30.76	2036.26	2036.26	2067.02	206.52	206.52	0.00	0.00	0.00	0.00	0.00
**H_2S (kg·d^{-1})**	0.10	0.10	0.00	0.00	0.10	0.00	0.00	0.00	0.00	0.00	0.00	0.00
**$NaNO_3$ (kg·d^{-1})**	0.00	0.00	0.00	0.00	0.00	0.00	0.00	271.18	0.00	0.00	0.00	0.00
**NaCl (kg·d^{-1})**	0.00	0.00	0.00	0.00	0.00	0.00	0.00	0.00	546.17	0.00	0.00	0.00
**Methanol (kg·d^{-1})**	0.00	0.00	0.00	0.00	0.00	0.00	0.00	0.00	0.00	0.00	0.00	0.00
**Na (kg·d^{-1})**	0.00	0.00	0.00	0.00	0.00	0.00	0.00	0.00	0.00	0.00	0.00	288.20
**Cl (kg·d^{-1})**	0.00	0.00	0.00	0.00	0.00	0.00	0.00	0.00	0.00	0.00	0.00	331.32
**NO_3- (kg·d^{-1})**	0.00	0.00	0.00	0.00	0.00	0.00	0.00	0.00	0.00	0.00	0.00	197.83
**NaOH (kg·d^{-1})**	0.00	0.00	0.00	0.00	0.00	0.00	0.00	0.00	0.00	0.00	0.00	0.00
**OH^- (kg·d^{-1})**	0.00	0.00	0.00	0.00	0.00	0.00	0.00	0.00	0.00	0.00	0.00	0.00
**H^+ (kg·d^{-1})**	0.00	0.00	0.00	0.00	0.00	0.00	0.00	0.00	0.00	0.00	0.00	0.00
**SO_4^{-2} (kg·d^{-1})**	0.00	0.00	0.00	0.00	0.00	0.00	0.00	0.00	0.00	0.00	0.00	0.00
**HCl (kg·d^{-1})**	0.00	0.00	0.00	0.00	0.00	0.00	0.00	0.00	0.00	0.00	0.00	0.00
**H_2SO_4 (kg·d^{-1})**	0.00	0.00	0.00	0.00	0.00	0.00	0.00	0.00	0.00	0.00	0.00	0.00
**Micronutrients (kg·d^{-1})**	0.00	0.00	0.00	0.00	0.00	0.00	0.00	0.00	0.00	2.89	0.00	2.89
**Water (kg·d^{-1})**	0.00	0.00	0.00	0.00	0.00	0.00	0.00	0.00	0.00	0.00	6621.14	6621.14
**Biomass (kg·d^{-1})**	0.00	0.00	0.00	0.00	0.00	0.00	0.00	0.00	0.00	0.00	0.00	0.00
**Ectoine (kg·d^{-1})**	0.00	0.00	0.00	0.00	0.00	0.00	0.00	0.00	0.00	0.00	0.00	0.00
**Na_2SO_4 (kg·d^{-1})**	0.00	0.00	0.00	0.00	0.00	0.00	0.00	0.00	0.00	0.00	0.00	0.00
**Others (kg·d^{-1})**	62.48	62.48	115.28	115.28	177.75	0.00	0.00	0.00	0.00	0.00	0.00	0.00

Table S1. Mass balance of the production of ectoine from biogas. In columns, the different streams according to Figures S1-S3. (Part 3/8).

**Stream**	**26**	**27**	**28**	**29**	**30**	**31**	**32**	**33**	**34**	**35**	**36**
**P (atm)**	3.71	1.00	1.00	1.00	3.71	1.00	1.15	1.00	1.15	1.00	1.00
**T (K)**	288.15	288.15	0.00	0.00	288.15	288.15	288.15	288.15	288.15	288.15	288.15
**Mass flow ($kg \cdot d^{-1}$)**	7441.37	76642.58	7114.91	64034.19	64034.19	5493.49	5493.49	20744.69	20744.69	26252.74	4917.37
**CH_4 ($kg \cdot d^{-1}$)**	0.00	0.00	0.00	0.00	0.00	0.00	0.00	0.00	0.00	0.00	0.00
**CO_2 ($kg \cdot d^{-1}$)**	0.00	49.23	4.62	41.62	41.62	2.98	2.98	0.00	0.00	17.54	2.83
**N_2 ($kg \cdot d^{-1}$)**	0.00	0.04	0.00	0.03	0.03	0.00	0.00	0.00	0.00	0.01	0.00
**O_2 ($kg \cdot d^{-1}$)**	0.00	0.00	0.00	0.00	0.00	0.00	0.00	0.00	0.00	0.00	0.00
**H_2S ($kg \cdot d^{-1}$)**	0.00	0.00	0.00	0.00	0.00	0.00	0.00	0.00	0.00	0.00	0.00
**$NaNO_3$ ($kg \cdot d^{-1}$)**	0.00	0.00	0.00	0.00	0.00	0.00	0.00	0.00	0.00	0.00	0.00
**NaCl ($kg \cdot d^{-1}$)**	0.00	0.00	0.00	0.00	0.00	0.00	0.00	0.00	0.00	0.00	0.00
**Methanol ($kg \cdot d^{-1}$)**	0.00	0.00	0.00	0.00	0.00	0.00	0.00	0.00	0.00	0.00	0.00
**Na ($kg \cdot d^{-1}$)**	288.20	1958.25	183.96	1655.68	1655.68	118.61	118.61	0.00	0.00	118.61	19.16
**Cl ($kg \cdot d^{-1}$)**	331.32	2251.28	211.49	1903.43	1903.43	136.36	136.36	0.00	0.00	136.36	22.02
**NO_3- ($kg \cdot d^{-1}$)**	197.83	38.43	3.61	32.50	32.50	2.33	2.33	0.00	0.00	2.33	0.38
**NaOH ($kg \cdot d^{-1}$)**	0.00	0.00	0.00	0.00	0.00	0.00	0.00	0.00	0.00	0.00	0.00
**OH^- ($kg \cdot d^{-1}$)**	0.00	0.00	0.00	0.00	0.00	0.00	0.00	0.00	0.00	0.00	0.00
**H^+ ($kg \cdot d^{-1}$)**	0.00	0.04	0.00	0.03	0.03	0.00	0.00	0.00	0.00	0.00	0.00
**SO_4^{-2} ($kg \cdot d^{-1}$)**	0.00	1.87	0.18	1.58	1.58	0.11	0.11	0.00	0.00	0.11	0.02
**HCl ($kg \cdot d^{-1}$)**	0.00	0.00	0.00	0.00	0.00	0.00	0.00	0.00	0.00	0.00	0.00
**H_2SO_4 ($kg \cdot d^{-1}$)**	0.00	0.00	0.00	0.00	0.00	0.00	0.00	0.00	0.00	0.00	0.00
**Micronutrients ($kg \cdot d^{-1}$)**	2.89	19.61	1.84	16.58	16.58	1.19	1.19	0.00	0.00	1.19	0.19
**Water ($kg \cdot d^{-1}$)**	6621.14	70158.80	6590.94	59318.47	59318.47	4249.38	4249.38	20744.69	20744.69	24994.07	4036.92
**Biomass ($kg \cdot d^{-1}$)**	0.00	894.61	4.47	40.26	40.26	849.88	849.88	0.00	0.00	849.88	807.38
**Ectoine ($kg \cdot d^{-1}$)**	0.00	62.62	0.31	2.82	2.82	59.49	59.49	0.00	0.00	59.49	16.64
**Na_2SO_4 ($kg \cdot d^{-1}$)**	0.00	0.00	0.00	0.00	0.00	0.00	0.00	0.00	0.00	0.00	0.00
**Others ($kg \cdot d^{-1}$)**	0.00	1207.81	113.47	1021.19	1021.19	73.15	73.15	0.00	0.00	73.15	11.82

Table S1. Mass balance of the production of ectoine from biogas. In columns, the different streams according to Figures S1-S3. (Part 4/8).

**Stream**	**37**	**38**	**39**	**40**	**41**	**42**	**43**	**44**	**45**	**46**	**47**
**P (atm)**	3.71	3.71	1.00	1.30	1.00	1.00	1.20	1.00	1.00	1.16	1.00
**T (K)**	288.15	288.15	288.15	288.15	288.15	288.15	288.15	288.15	288.15	288.15	288.15
**Mass flow (kg·d^{-1})**	1227.45	3689.92	21335.38	21335.38	2171.36	19164.02	19164.02	2084.23	17079.79	17079.79	19.32
**CH_4 (kg·d^{-1})**	0.00	0.00	0.00	0.00	0.00	0.00	0.00	0.00	0.00	0.00	0.00
**CO_2 (kg·d^{-1})**	0.71	2.13	14.71	14.71	1.47	13.23	13.23	1.32	11.91	11.91	0.00
**N_2 (kg·d^{-1})**	0.00	0.00	0.01	0.01	0.00	0.01	0.01	0.00	0.01	0.01	0.00
**O_2 (kg·d^{-1})**	0.00	0.00	0.00	0.00	0.00	0.00	0.00	0.00	0.00	0.00	0.00
**H_2S (kg·d^{-1})**	0.00	0.00	0.00	0.00	0.00	0.00	0.00	0.00	0.00	0.00	0.00
**$NaNO_3$ (kg·d^{-1})**	0.00	0.00	0.00	0.00	0.00	0.00	0.00	0.00	0.00	0.00	0.00
**NaCl (kg·d^{-1})**	0.00	0.00	0.00	0.00	0.00	0.00	0.00	0.00	0.00	0.00	0.00
**Methanol (kg·d^{-1})**	0.00	0.00	0.00	0.00	0.00	0.00	0.00	0.00	0.00	0.00	0.00
**Na (kg·d^{-1})**	4.78	14.38	99.45	99.45	9.95	89.51	89.51	85.93	3.58	3.58	0.00
**Cl (kg·d^{-1})**	5.50	16.53	114.33	114.33	11.43	102.90	102.90	98.78	4.12	4.12	0.00
**NO_3- (kg·d^{-1})**	0.09	0.28	1.95	1.95	0.20	1.76	1.76	1.69	0.07	0.07	0.00
**NaOH (kg·d^{-1})**	0.00	0.00	0.00	0.00	0.00	0.00	0.00	0.00	0.00	0.00	0.00
**OH^- (kg·d^{-1})**	0.00	0.00	0.00	0.00	0.00	0.00	0.00	0.00	0.00	0.00	0.00
**H^+ (kg·d^{-1})**	0.00	0.00	0.00	0.00	0.00	0.00	0.00	0.00	0.00	0.00	0.00
**SO_4^{-2} (kg·d^{-1})**	0.00	0.01	0.10	0.10	0.01	0.09	0.09	0.08	0.00	0.00	0.00
**HCl (kg·d^{-1})**	0.00	0.00	0.00	0.00	0.00	0.00	0.00	0.00	0.00	0.00	6.20
**H_2SO_4 (kg·d^{-1})**	0.00	0.00	0.00	0.00	0.00	0.00	0.00	0.00	0.00	0.00	0.00
**Micronutrients (kg·d^{-1})**	0.05	0.14	1.00	1.00	0.10	0.90	0.90	0.86	0.04	0.04	0.00
**Water (kg·d^{-1})**	1007.68	3029.24	20957.16	20957.16	2095.72	18861.44	18861.44	1886.14	16975.30	16975.30	13.13
**Biomass (kg·d^{-1})**	201.54	605.85	42.49	42.49	42.07	0.42	0.42	0.04	0.38	0.38	0.00
**Ectoine (kg·d^{-1})**	4.15	12.49	42.85	42.85	4.28	38.56	38.56	3.86	34.71	34.71	0.00
**Na_2SO_4 (kg·d^{-1})**	0.00	0.00	0.00	0.00	0.00	0.00	0.00	0.00	0.00	0.00	0.00
**Others (kg·d^{-1})**	2.95	8.87	61.34	61.34	6.13	55.21	55.21	5.52	49.68	49.68	0.00

Table S1. Mass balance of the production of ectoine from biogas. In columns, the different streams according to Figures S1-S3. (Part 5/8).

**Stream**	**48**	**49**	**50**	**51**	**52**	**53**	**54**	**55**	**56**	**57**	**58**
**P (atm)**	1.16	1.00	1.39	1.00	1.00	1.00	1.00	1.39	1.00	1.00	1.39
**T (K)**	288.15	288.15	288.15	288.15	288.15	288.15	288.15	288.15	288.15	288.15	288.15
**Mass flow (kg·d^{-1})**	19.32	17099.12	17099.12	17067.88	40.64	755.86	796.49	796.49	796.49	780.88	780.88
**CH_4 (kg·d^{-1})**	0.00	0.00	0.00	0.00	0.00	0.00	0.00	0.00	0.00	0.00	0.00
**CO_2 (kg·d^{-1})**	0.00	11.91	11.91	11.91	0.00	0.00	0.00	0.00	0.00	0.00	0.00
**N_2 (kg·d^{-1})**	0.00	0.01	0.01	0.01	0.00	0.00	0.00	0.00	0.00	0.00	0.00
**O_2 (kg·d^{-1})**	0.00	0.00	0.00	0.00	0.00	0.00	0.00	0.00	0.00	0.00	0.00
**H_2S (kg·d^{-1})**	0.00	0.00	0.00	0.00	0.00	0.00	0.00	0.00	0.00	0.00	0.00
**$NaNO_3$ (kg·d^{-1})**	0.00	0.00	0.00	0.00	0.00	0.00	0.00	0.00	0.00	0.00	0.00
**NaCl (kg·d^{-1})**	0.00	0.00	0.00	0.00	0.00	0.00	0.00	0.00	0.00	0.00	0.00
**Methanol (kg·d^{-1})**	0.00	0.00	0.00	0.00	0.00	0.00	0.00	0.00	0.00	0.00	0.00
**Na (kg·d^{-1})**	0.00	3.58	3.58	3.58	0.00	0.00	0.00	0.00	0.00	0.00	0.00
**Cl (kg·d^{-1})**	0.00	4.12	4.12	4.12	0.00	0.00	0.00	0.00	0.00	0.00	0.00
**NO_3- (kg·d^{-1})**	0.00	0.07	0.07	0.07	0.00	0.00	0.00	0.00	0.00	0.00	0.00
**NaOH (kg·d^{-1})**	0.00	0.00	0.00	0.00	0.00	0.00	0.00	0.00	0.00	0.00	0.00
**OH^- (kg·d^{-1})**	0.00	0.00	0.00	0.00	0.00	0.00	0.00	0.00	0.00	0.00	0.00
**H^+ (kg·d^{-1})**	0.00	0.00	0.00	0.00	0.00	0.00	0.00	0.00	0.00	0.00	0.00
**SO_4^{-2} (kg·d^{-1})**	0.00	0.00	0.00	0.00	0.00	0.00	0.00	0.00	0.00	0.00	0.00
**HCl (kg·d^{-1})**	6.20	6.20	6.20	6.20	0.00	0.00	0.00	0.00	0.00	0.00	0.00
**H_2SO_4 (kg·d^{-1})**	0.00	0.00	0.00	0.00	39.82	0.00	39.82	39.82	39.82	0.00	0.00
**Micronutrients (kg·d^{-1})**	0.00	0.04	0.04	0.04	0.00	0.00	0.00	0.00	0.00	0.00	0.00
**Water (kg·d^{-1})**	13.13	16988.42	16988.42	16988.42	0.81	755.86	756.67	756.67	756.67	780.88	780.88
**Biomass (kg·d^{-1})**	0.00	0.38	0.38	0.38	0.00	0.00	0.00	0.00	0.00	0.00	0.00
**Ectoine (kg·d^{-1})**	0.00	34.71	34.71	3.47	0.00	0.00	0.00	0.00	0.00	0.00	0.00
**Na_2SO_4 (kg·d^{-1})**	0.00	0.00	0.00	0.00	0.00	0.00	0.00	0.00	0.00	0.00	0.00
**Others (kg·d^{-1})**	0.00	49.68	49.68	49.68	0.00	0.00	0.00	0.00	0.00	0.00	0.00

Table S1. Mass balance of the production of ectoine from biogas. In columns, the different streams according to Figures S1-S3. (Part 6/8).

Stream	59	60	61	62	63	64	65	66	67	68	69
P (atm)	1.00	1.00	1.00	1.00	1.39	1.00	1.00	1.05	1.00	1.05	1.00
T (K)	288.15	288.15	288.15	288.15	288.15	288.15	288.15	288.15	288.15	288.15	288.15
Mass flow ($kg \cdot d^{-1}$)	780.88	2292.26	120.65	2412.91	2412.91	1608.60	835.54	835.54	49.20	49.20	884.74
CH_4 ($kg \cdot d^{-1}$)	0.00	0.00	0.00	0.00	0.00	0.00	0.00	0.00	0.00	0.00	0.00
CO_2 ($kg \cdot d^{-1}$)	0.00	0.00	0.00	0.00	0.00	0.00	0.00	0.00	0.00	0.00	0.00
N_2 ($kg \cdot d^{-1}$)	0.00	0.00	0.00	0.00	0.00	0.00	0.00	0.00	0.00	0.00	0.00
O_2 ($kg \cdot d^{-1}$)	0.00	0.00	0.00	0.00	0.00	0.00	0.00	0.00	0.00	0.00	0.00
H_2S ($kg \cdot d^{-1}$)	0.00	0.00	0.00	0.00	0.00	0.00	0.00	0.00	0.00	0.00	0.00
$NaNO_3$ ($kg \cdot d^{-1}$)	0.00	0.00	0.00	0.00	0.00	0.00	0.00	0.00	0.00	0.00	0.00
NaCl ($kg \cdot d^{-1}$)	0.00	0.00	0.00	0.00	0.00	0.00	0.00	0.00	0.00	0.00	0.00
Methanol ($kg \cdot d^{-1}$)	0.00	0.00	0.00	0.00	0.00	0.00	0.00	0.00	0.00	0.00	0.00
Na ($kg \cdot d^{-1}$)	0.00	0.00	0.00	0.00	0.00	0.00	0.00	0.00	0.00	0.00	0.51
Cl ($kg \cdot d^{-1}$)	0.00	0.00	0.00	0.00	0.00	0.00	0.00	0.00	0.00	0.00	0.00
NO_3- ($kg \cdot d^{-1}$)	0.00	0.00	0.00	0.00	0.00	0.00	0.00	0.00	0.00	0.00	0.00
NaOH ($kg \cdot d^{-1}$)	0.00	0.00	120.65	120.65	120.65	80.43	40.22	40.22	0.00	0.00	0.00
OH^- ($kg \cdot d^{-1}$)	0.00	0.00	0.00	0.00	0.00	0.00	0.00	0.00	0.00	0.00	0.38
H^+ ($kg \cdot d^{-1}$)	0.00	0.00	0.00	0.00	0.00	0.00	0.00	0.00	0.00	0.00	0.00
SO_4^{-2} ($kg \cdot d^{-1}$)	0.00	0.00	0.00	0.00	0.00	0.00	0.00	0.00	0.00	0.00	0.00
HCl ($kg \cdot d^{-1}$)	0.00	0.00	0.00	0.00	0.00	0.00	0.00	0.00	0.00	0.00	0.00
H_2SO_4 ($kg \cdot d^{-1}$)	0.00	0.00	0.00	0.00	0.00	0.00	0.00	0.00	48.22	48.22	0.00
Micronutrients ($kg \cdot d^{-1}$)	0.00	0.00	0.00	0.00	0.00	0.00	0.00	0.00	0.00	0.00	0.00
Water ($kg \cdot d^{-1}$)	780.88	2292.26	0.00	2292.26	2292.26	1528.17	764.09	764.09	0.98	0.98	782.79
Biomass ($kg \cdot d^{-1}$)	0.00	0.00	0.00	0.00	0.00	0.00	0.00	0.00	0.00	0.00	0.00
Ectoine ($kg \cdot d^{-1}$)	0.00	0.00	0.00	0.00	0.00	0.00	31.24	31.24	0.00	0.00	31.24
Na_2SO_4 ($kg \cdot d^{-1}$)	0.00	0.00	0.00	0.00	0.00	0.00	0.00	0.00	0.00	0.00	69.83
Others ($kg \cdot d^{-1}$)	0.00	0.00	0.00	0.00	0.00	0.00	0.00	0.00	0.00	0.00	0.00

Table S1. Mass balance of the production of ectoine from biogas. In columns, the different streams according to Figures S1-S3. (Part 7/8).

**Stream**	**70**	**71**	**72**	**73**	**74**	**75**	**76**	**77**	**78**	**79**	**80**	**81**
**P (atm)**	1.30	1.00	1.30	1.00	1.00	1.00	1.03	1.00	1.30	1.00	1.00	1.09
**T (K)**	288.15	288.15	-	288.15	288.15	288.15	288.15	288.15	288.15	288.15	288.15	288.15
**Mass flow (kg·d^{-1})**	884.74	52076.09	52076.09	52857.23	103.60	312.35	312.35	415.95	415.95	103.71	312.24	312.24
**CH_4 (kg·d^{-1})**	0.00	0.00	0.00	0.00	0.00	0.00	0.00	0.00	0.00	0.00	0.00	0.00
**CO_2 (kg·d^{-1})**	0.00	24.85	24.85	24.85	0.00	0.00	0.00	0.00	0.00	0.00	0.00	0.00
**N_2 (kg·d^{-1})**	0.00	39285.64	39285.64	39285.64	0.00	0.00	0.00	0.00	0.00	0.00	0.00	0.00
**O_2 (kg·d^{-1})**	0.00	12081.65	12081.65	12081.65	0.00	0.00	0.00	0.00	0.00	0.00	0.00	0.00
**H_2S (kg·d^{-1})**	0.00	0.00	0.00	0.00	0.00	0.00	0.00	0.00	0.00	0.00	0.00	0.00
**$NaNO_3$ (kg·d^{-1})**	0.00	0.00	0.00	0.00	0.00	0.00	0.00	0.00	0.00	0.00	0.00	0.00
**NaCl (kg·d^{-1})**	0.00	0.00	0.00	0.00	0.00	0.00	0.00	0.00	0.00	0.00	0.00	0.00
**Methanol (kg·d^{-1})**	0.00	0.00	0.00	0.00	0.00	312.35	312.35	312.35	312.35	31.24	281.12	281.12
**Na (kg·d^{-1})**	0.51	0.00	0.00	0.00	0.51	0.00	0.00	0.51	0.51	0.05	0.46	0.46
**Cl (kg·d^{-1})**	0.00	0.00	0.00	0.00	0.00	0.00	0.00	0.00	0.00	0.00	0.00	0.00
**NO_3- (kg·d^{-1})**	0.00	0.00	0.00	0.00	0.00	0.00	0.00	0.00	0.00	0.00	0.00	0.00
**NaOH (kg·d^{-1})**	0.00	0.00	0.00	0.00	0.00	0.00	0.00	0.00	0.00	0.00	0.00	0.00
**OH^- (kg·d^{-1})**	0.38	0.00	0.00	0.00	0.38	0.00	0.00	0.38	0.38	0.00	0.38	0.38
**H^+ (kg·d^{-1})**	0.00	0.00	0.00	0.00	0.00	0.00	0.00	0.00	0.00	0.00	0.00	0.00
**SO_4^{-2} (kg·d^{-1})**	0.00	0.00	0.00	0.00	0.00	0.00	0.00	0.00	0.00	0.00	0.00	0.00
**HCl (kg·d^{-1})**	0.00	0.00	0.00	0.00	0.00	0.00	0.00	0.00	0.00	0.00	0.00	0.00
**H_2SO_4 (kg·d^{-1})**	0.00	0.00	0.00	0.00	0.00	0.00	0.00	0.00	0.00	0.00	0.00	0.00
**Micronutrients (kg·d^{-1})**	0.00	0.00	0.00	0.00	0.00	0.00	0.00	0.00	0.00	0.00	0.00	0.00
**Water (kg·d^{-1})**	782.79	0.00	0.00	781.14	1.64	0.00	0.00	1.64	1.64	0.16	1.48	1.48
**Biomass (kg·d^{-1})**	0.00	0.00	0.00	0.00	0.00	0.00	0.00	0.00	0.00	0.00	0.00	0.00
**Ectoine (kg·d^{-1})**	31.24	0.00	0.00	0.00	31.24	0.00	0.00	31.24	31.24	3.12	28.11	28.11
**Na_2SO_4 (kg·d^{-1})**	69.83	0.00	0.00	0.00	69.83	0.00	0.00	69.83	69.83	69.13	0.70	0.70
**Others (kg·d^{-1})**	0.00	683.96	683.96	683.96	0.00	0.00	0.00	0.00	0.00	0.00	0.00	0.00

Table S1. Mass balance of the production of ectoine from biogas. In columns, the different streams according to Figures S1-S3. (Part 8/8).

**Stream**	**82**	**83**	**84**	**85**	**86**	**87**	**88**	**89**	**90**	**91**	**92**
**P (atm)**	1.00	1.00	1.00	1.10	1.00	1.00	1.20	1.00	1.20	1.00	1.00
**T (K)**	337.85	288.15	288.15	288.15	288.15	288.15	288.15	288.15	-	288.15	288.15
**Mass flow (kg·d^{-1})**	278.30	278.30	33.94	33.94	3.76	30.17	30.17	58.69	58.69	61.46	27.40
**CH_4 (kg·d^{-1})**	0.00	0.00	0.00	0.00	0.00	0.00	0.00	0.00	0.00	0.00	0.00
**CO_2 (kg·d^{-1})**	0.00	0.00	0.00	0.00	0.00	0.00	0.00	0.03	0.03	0.03	0.00
**N_2 (kg·d^{-1})**	0.00	0.00	0.00	0.00	0.00	0.00	0.00	44.27	44.27	44.27	0.00
**O_2 (kg·d^{-1})**	0.00	0.00	0.00	0.00	0.00	0.00	0.00	13.62	13.62	13.62	0.00
**H_2S (kg·d^{-1})**	0.00	0.00	0.00	0.00	0.00	0.00	0.00	0.00	0.00	0.00	0.00
**$NaNO_3$ (kg·d^{-1})**	0.00	0.00	0.00	0.00	0.00	0.00	0.00	0.00	0.00	0.00	0.00
**NaCl (kg·d^{-1})**	0.00	0.00	0.00	0.00	0.00	0.00	0.00	0.00	0.00	0.00	0.00
**Methanol (kg·d^{-1})**	278.30	278.30	2.81	2.81	1.14	1.67	1.67	0.00	0.00	1.67	0.00
**Na (kg·d^{-1})**	0.00	0.00	0.46	0.46	0.19	0.27	0.27	0.00	0.00	0.00	0.27
**Cl (kg·d^{-1})**	0.00	0.00	0.00	0.00	0.00	0.00	0.00	0.00	0.00	0.00	0.00
**NO_3- (kg·d^{-1})**	0.00	0.00	0.00	0.00	0.00	0.00	0.00	0.00	0.00	0.00	0.00
**NaOH (kg·d^{-1})**	0.00	0.00	0.00	0.00	0.00	0.00	0.00	0.00	0.00	0.00	0.00
**OH^- (kg·d^{-1})**	0.00	0.00	0.38	0.38	0.15	0.22	0.22	0.00	0.00	0.22	0.00
**H^+ (kg·d^{-1})**	0.00	0.00	0.00	0.00	0.00	0.00	0.00	0.00	0.00	0.00	0.00
**SO_4^{-2} (kg·d^{-1})**	0.00	0.00	0.00	0.00	0.00	0.00	0.00	0.00	0.00	0.00	0.00
**HCl (kg·d^{-1})**	0.00	0.00	0.00	0.00	0.00	0.00	0.00	0.00	0.00	0.00	0.00
**H_2SO_4 (kg·d^{-1})**	0.00	0.00	0.00	0.00	0.00	0.00	0.00	0.00	0.00	0.00	0.00
**Micronutrients (kg·d^{-1})**	0.00	0.00	0.00	0.00	0.00	0.00	0.00	0.00	0.00	0.00	0.00
**Water (kg·d^{-1})**	0.00	0.00	1.48	1.48	0.60	0.88	0.88	0.00	0.00	0.88	0.00
**Biomass (kg·d^{-1})**	0.00	0.00	0.00	0.00	0.00	0.00	0.00	0.00	0.00	0.00	0.00
**Ectoine (kg·d^{-1})**	0.00	0.00	28.11	28.11	1.41	26.71	26.71	0.00	0.00	0.00	26.71
**Na_2SO_4 (kg·d^{-1})**	0.00	0.00	0.70	0.70	0.28	0.42	0.42	0.00	0.00	0.00	0.42
**Others (kg·d^{-1})**	0.00	0.00	0.00	0.00	0.00	0.00	0.00	0.77	0.77	0.77	0.00

Table S2. Mass balance of the ectoine biosynthesis from biogas. The mass balance includes Streams 1-41.

Substance	Mass in ($kg \cdot d^{-1}$)	Mass out ($kg \cdot d^{-1}$)	Mass balance ($kg \cdot d^{-1}$)
**Global**	40227.6	40227.6	**0.0**
**CH_4**	690.3	69.0	621.3
**CO_2**	1108.8	2290.5	-1181.7
**N_2**	6671.5	6680.9	-9.4
**O_2**	2047.7	206.5	1841.2
**H_2S**	9.8	0.0	9.8
**$NaNO_3$**	331.5	0.0	331.5
**NaCl**	546.2	0.0	546.2
**Methanol**	0.0	0.0	0.0
**Na^+**	0.0	311.0	-311.0
**Cl^-**	0.0	331.3	-331.3
**NO_3^-**	0.0	5.7	-5.7
**NaOH**	11.3	0.0	11.3
**OH^-**	0.0	0.0	0.0
**H^+**	0.0	0.0	0.0
**SO_4^{-2}**	0.0	27.6	-27.6
**S^0**	0.0	0.0	0.0
**HCl**	0.0	0.0	0.0
**H_2SO_4**	0.0	0.0	0.0
**Micronutrients**	3.4	3.4	0.0
**Water**	28629.2	29824.3	-1195.0
**Biomass**	0.0	252.1	-252.1
**Ectoine**	0.0	47.3	-47.3
**Others**	177.8	177.8	0.0

Table S3. Mass balance of the ectoine extraction and purification. The mass balance includes Streams 41-92.

Substance	Mass in ($kg \cdot d^{-1}$)	Mass out ($kg \cdot d^{-1}$)	Mass balance ($kg \cdot d^{-1}$)
**Global**	77841.3	77841.3	**0.0**
**CH_4**	0.0	0.0	0.0
**CO_2**	39.6	39.6	0.0
**N_2**	39329.9	39329.9	0.0
**O_2**	12095.3	12095.3	0.0
**H_2S**	0.0	0.0	0.0
**$NaNO_3$**	0.0	0.0	0.0
**NaCl**	0.0	0.0	0.0
**Methanol**	312.4	312.4	0.0
**Na^+**	99.5	100.0	-0.5
**Cl^-**	114.3	114.3	0.0
**NO_3^-**	2.0	2.0	0.0
**NaOH**	120.6	80.4	40.2
**OH^-**	0.0	0.4	-0.4
**H^+**	0.0	0.0	0.0
**SO_4^{-2}**	0.1	0.1	0.0
**S^0**	0.0	0.0	0.0
**HCl**	6.2	6.2	0.0
**H_2SO_4**	88.0	39.8	48.2
**Micronutrients**	1.0	1.0	0.0
**Water**	24801.1	24818.8	-17.7
**Biomass**	42.5	42.5	0.0
**Ectoine**	42.8	42.8	0.0
**Na_2SO_4**	0.0	69.8	-69.8
**Others**	746.1	746.1	0.0

Table S4. Elemental mass balance of the ectoine biosynthesis from biogas. The mass balance includes Streams 1-41.

Element ($kmol\cdot d^{-1}$)	C	N	O	H	S	Na	Cl
**Global**	**0.00**	**0.00**	**0.00**	**0.00**	**0.00**	**0.00**	**0.00**
**CH_4**	38.73	0.00	0.00	154.90	0.00	0.00	0.00
**CO_2**	-26.85	0.00	-53.70	0.00	0.00	0.00	0.00
**N_2**	0.00	-0.67	0.00	0.00	0.00	0.00	0.00
**O_2**	0.00	0.00	115.08	0.00	0.00	0.00	0.00
**H_2S**	0.00	0.00	0.00	0.57	0.29	0.00	0.00
**$NaNO_3$**	0.00	3.90	11.70	0.00	0.00	3.90	0.00
**NaCl**	0.00	0.00	0.00	0.00	0.00	9.35	9.35
**CH_3OH**	0.00	0.00	0.00	0.00	0.00	0.00	0.00
**Na^+**	0.00	0.00	0.00	0.00	0.00	-13.53	0.00
**Cl^-**	0.00	0.00	0.00	0.00	0.00	0.00	-9.35
**NO_3^-**	0.00	-0.09	-0.28	0.00	0.00	0.00	0.00
**NaOH**	0.00	0.00	0.28	0.28	0.00	0.28	0.00
**OH^-**	0.00	0.00	0.00	0.00	0.00	0.00	0.00
**H^+**	0.00	0.00	0.00	-0.01	0.00	0.00	0.00
**SO_4^{-2}**	0.00	0.00	-1.15	0.00	-0.29	0.00	0.00
**S^0**	0.00	0.00	0.00	0.00	0.00	0.00	0.00
**HCl**	0.00	0.00	0.00	0.00	0.00	0.00	0.00
**H_2SO_4**	0.00	0.00	0.00	0.00	0.00	0.00	0.00
**Micronutrients**	0.00	0.00	0.00	0.00	0.00	0.00	0.00
**Water**	0.00	0.00	-66.33	-132.67	0.00	0.00	0.00
**Biomass**	-9.88	-2.47	-4.94	-19.75	0.00	0.00	0.00
**Ectoine**	-2.00	-0.67	-0.67	-3.33	0.00	0.00	0.00
**Others**	0.00	0.00	0.00	0.00	0.00	0.00	0.00

Table S5. Elemental mass balance of the ectoine extraction and purification. The mass balance includes Streams 41-92.

Element (kmol·d^{-1})	C	N	O	H	S	Na	Cl
**Global**	0.00	0.00	0.00	0.00	0.00	0.00	0.00
**CH_4**	0.00	0.00	0.00	0.00	0.00	0.00	0.00
**CO_2**	0.00	0.00	0.00	0.00	0.00	0.00	0.00
**N_2**	0.00	0.00	0.00	0.00	0.00	0.00	0.00
**O_2**	0.00	0.00	0.00	0.00	0.00	0.00	0.00
**H_2S**	0.00	0.00	0.00	0.00	0.00	0.00	0.00
**$NaNO_3$**	0.00	0.00	0.00	0.00	0.00	0.00	0.00
**NaCl**	0.00	0.00	0.00	0.00	0.00	0.00	0.00
**CH_3OH**	0.00	0.00	0.00	0.00	0.00	0.00	0.00
**Na^+**	0.00	0.00	0.00	0.00	0.00	-0.02	0.00
**Cl^-**	0.00	0.00	0.00	0.00	0.00	0.00	0.00
**NO_3^-**	0.00	0.00	0.00	0.00	0.00	0.00	0.00
**NaOH**	0.00	0.00	1.01	1.01	0.00	1.01	0.00
**OH^-**	0.00	0.00	-0.02	-0.02	0.00	0.00	0.00
**H^+**	0.00	0.00	0.00	0.00	0.00	0.00	0.00
**SO_4^{-2}**	0.00	0.00	0.00	0.00	0.00	0.00	0.00
**S^0**	0.00	0.00	0.00	0.00	0.00	0.00	0.00
**HCl**	0.00	0.00	0.00	0.00	0.00	0.00	0.00
**H_2SO_4**	0.00	0.00	1.97	0.98	0.49	0.00	0.00
**Micronutrients**	0.00	0.00	0.00	0.00	0.00	0.00	0.00
**Water**	0.00	0.00	-0.98	-1.97	0.00	0.00	0.00
**Biomass**	0.00	0.00	0.00	0.00	0.00	0.00	0.00
**Ectoine**	0.00	0.00	0.00	0.00	0.00	0.00	0.00
**Na_2SO_4**	0.00	0.00	-1.97	0.00	-0.49	-0.98	0.00
**Others**	0.00	0.00	0.00	0.00	0.00	0.00	0.00

Table S6. Summary of purchased equipment cost and total investment cost.

Nº	Description	Volume (L)	Flow ($m^3 \cdot d^{-1}$)	Price (€)
**E-01**	Biogas blower	-	1695.70	2,165
**E-02**	Biotrickling filter	4019	-	53,049
**E-03**	Nutrients pump	-	1.21	373
**E-04**	NaOH pump	-	0.06	79
**E-05**	Trickling pump	-	312.24	6,319
**E-06**	Biogas blower	-	1707.79	2,178
**E-07**	Air blower	-	7165.03	6,758
**E-08**	Bubble column bioreactor	194886	-	584,657
**E-10**	Nutrients pump	-	6.62	888
**E-11**	Biomass centrifuge	-	70.16	11,744
**E-12**	Biomass recirculation pump	-	59.32	2,712
**E-13**	Biomass pump	-	4.25	709
**E-14**	Water pump	-	20.74	1,589
**E-15**	Ectoine extraction CSTR	104	-	5,663
**E-16**	Ectoine centrifuge	-	24.99	6,633
**E-17**	Liquid recirculation pump	-	4.04	690
**E-18**	Ectoine pump	-	20.96	1,597
**E-19**	Ultrafiltration Membrane	-	20.96	11,643
**E-20**	Permeate pump	-	18.86	1,513
**E-21**	Electrodialysis cell	-	18.86	15,718
**E-22**	Permeate pump	-	16.98	1,434
**E-23**	HCl pump	-	0.02	42
**E-24**	Acidification CSTR	1699	-	24,833
**E-25**	Ectoine IEX pump	-	16.99	1,435
**E-26**	Ionic Exchange Column	500	-	28,763
**E-27**	H_2SO_4 pump	-	0.78	299
**E-28**	Distilled water pump	-	0.78	299
**E-29**	NaOH pump	-	2.34	523
**E-30**	Ectoine pump	-	0.78	299
**E-31**	H_2SO_4 pump	-	0.03	54
**E-32**	Neutralization CSTR	40	-	3,430
**E-33**	Spray drying pump	-	0.81	304
**E-34**	Air blower	-	42511.85	27,572
**E-35**	Spray dryer	369	-	177,035
**E-36**	Methanol pump	-	0.39	211
**E-37**	Methanol solubilisation CSTR	20	-	2,347
**E-38**	Ectoine in methanol pump	-	0.39	211
**E-39**	Ultrafiltration membrane	-	0.39	219
**E-40**	Permeate pump	-	0.30	183
**E-41**	Ectoine crystallizer	15	0.00	12,173
**E-42**	Methanol condenser	-	0.30	13,160

**E-43**	Ectoine concentrate pump	-	0.03	61
**E-44**	Ectoine centrifuge	-	0.03	172
**E-46**	Air blower	-	47.91	130
**E-47**	Tray dryer	-	0.03	16,838
	**Total PEC**			**1,028,704 €**

Table S7 Summary of individual equipment energy and power consumption.

Nº	Description	Operation time ($h{\cdot}d^{-1}$)	Power consumption (W)	Energy consumption ($kWh{\cdot}d^{-1}$)
**E-01**	Biogas blower	24	466.36	11.19
**E-03**	Nutrients pump	24	0.20	0.005
**E-04**	NaOH pump	24	0.01	0.0002
**E-05**	Trickling pump	24	259.69	6.23
**E-06**	Biogas blower	24	4456.91	106.97
**E-07**	Air blower	24	19456.63	466.96
**E-10**	Nutrients pump	24	30.11	0.72
**E-11**	Biomass centrifuge	24	2923.28	70.16
**E-12**	Biomass recirculation pump	24	269.72	6.47
**E-13**	Biomass pump	24	1.04	0.03
**E-14**	Water pump	24	5.09	0.12
**E-15**	Ectoine extraction CSTR	24	86.78	2.08
**E-16**	Ectoine centrifuge	24	1041.42	24.99
**E-17**	Liquid recirculation pump	24	18.36	0.44
**E-18**	Ectoine pump	24	10.53	0.25
**E-20**	Permeate pump	24	6.32	0.15
**E-21**	Electrodialysis cell	24	5501.25	132.03
**E-22**	Permeate pump	24	4.66	0.11
**E-23**	HCl pump	24	0.005	0.0001
**E-24**	Acidification CSTR	24	1416.00	33.98
**E-25**	Ectoine IEX pump	24	11.01	0.26
**E-27**	H2SO4 pump	24	0.51	0.01
**E-28**	Distilled water pump	24	0.51	0.01
**E-29**	NaOH pump	24	1.52	0.04
**E-30**	Ectoine pump	24	0.06	0.001
**E-31**	H2SO4 pump	24	0.002	0.0001
**E-32**	Neutralization CSTR	24	40.38	0.97
**E-33**	Spray drying pump	24	0.41	0.01
**E-34**	Air blower	24	21366.64	512.80
**E-36**	Methanol pump	24	0.02	0.0004
**E-37**	Methanol solubilization CSTR	24	16.43	0.39
**E-38**	Ectoine in methanol pump	24	0.20	0.005
**E-40**	Permeate pump	24	0.04	0.001
**E-41**	Ectoine crystallizer	24	12.34	0.30
**E-43**	Ectoine concentrate pump	24	0.01	0.00
**E-44**	Ectoine centrifuge	24	1.41	0.03
**E-46**	Air blower	24	16.05	0.39
**Total Energy consumption ($kWh{\cdot}d^{-1}$)**		-	-	1,797.85

List of equations used in the design and dimensioning of the equipment:

1. Bubble column bioreactor

$$\mathrm{CH_4} + \frac{3}{8}\mathrm{O_2} + \frac{1}{4}\mathrm{NO_3^-} \rightarrow \frac{1}{4}\mathrm{C_4H_8O_2N} + \mathrm{H_2O} \tag{S1}$$

$$\mathrm{CH_4} + \frac{1}{4}\mathrm{O_2} + \frac{1}{3}\mathrm{NO_3^-} \rightarrow \frac{7}{6}\mathrm{H_2O} + \frac{1}{6}\mathrm{C_6H_{10}N_2O_2} \tag{S2}$$

$$\mathrm{CH_4} + 2\,\mathrm{O_2} \rightarrow 2\,\mathrm{H_2O} + \mathrm{CO_2} \tag{S3}$$

$$CH_4 - RE = \frac{Q_{in} \cdot Y_{CH_{4_{in}}} - Q_{out} \cdot Y_{CH_{4_{out}}}}{Q_{in} \cdot Y_{CH_{4_{in}}}} \tag{S4}$$

$$CH_4 - EC = \frac{Q_{in} \cdot Y_{CH_{4_{in}}} - Q_{out} \cdot Y_{CH_{4_{out}}}}{V} \tag{S5}$$

$$kla_{CO} = \frac{CO - EC}{\left(\frac{C_{CO,in}}{H_{CO}} - C_{L,CO}\right)} \tag{S6}$$

$$\frac{kla_{CH_4}}{kla_{CO}} = \frac{\left(\frac{1}{V_{m,\,CH_4}}\right)^{0.4}}{\left(\frac{1}{V_{m,\,CO}}\right)^{0.4}} \tag{S7}$$

$$CH_4 - EC = kla_{CH_4} \cdot \left(\frac{C_{CH_4,in}}{H_{CH_4}} - C_{L,CH_4}\right) \tag{S8}$$

$$EBRT = \frac{V}{Q_{in}} \tag{S9}$$

$$V = \frac{\pi \cdot D^2}{4} \cdot H \tag{S10}$$

$$\frac{H}{D} = 10 \tag{S11}$$

$$Ht = 1.1 \cdot H \tag{S12}$$

$$V_r = \frac{\pi \cdot D^2}{4} \cdot Ht \tag{S13}$$

Where:

CH_4-RE = Methane removal efficiency.

Q_{in} = Inlet volumetric gas flow.

$Y_{CH4,in}$ = Methane gas molar fraction of the inlet flow.

Q_{out} = Outlet volumetric gas flow.

$Y_{CH4,out}$ = Methane gas molar fraction of the outlet flow.

CH_4-EC = Methane elimination capacity.

V = Liquid volume in the bubble column bioreactor.

kla_{CO} = Volumetric mass transfer coefficient of carbon monoxide.

CO –EC = Carbon monoxide elimination capacity.

$C_{CO,in}$ = Inlet carbon monoxide gas concentration.

H_{CO} = Carbon monoxide Henry’s law dimensionless constant.

$C_{l,,CO}$ = Bulk aqueous concentration of carbon monoxide.

kla_{CH4} = Volumetric mass transfer coefficient of methane.

Vm_{CH4} = Molar volume at the normal boiling point of methane.

Vm_{CO} = Molar volume at the normal boiling point of carbon monoxide.

$C_{CH4,in}$ = Inlet methane gas concentration.

$C_{L,CH4}$ = Bulk aqueous concentration of methane.

H_{CH4} = Methane Henry's law dimensionless constant.

EBRT = Gas residence time in the bubble column bioreactor.

D = Bubble column bioreactor diameter.

H = Liquid height in the bubble column bioreactor.

H_t = Total height of the bubble column bioreactor, including overhead.

V_r = Total volume of the bubble column bioreactor.

The calculation algorithm is described as follows:

1. Q_{biogas} and Q_{air} are calculated from the mass balance (Streams 17 and 19).
2. Assuming a CH_4-*RE* of 90%, Q_{out} and $Y_{CH4,out}$ are calculated with equations S1-S4.
3. It is necessary to calculate previously CH_4-*EC* in order to estimate *V* with equation S5.
4. Known *CO-EC*, $C_{CO,in}$, $C_{L,CO}$ and H_{CO}, kla_{CO} is calculated with equation S6.
5. Known kla_{CO}, Vm_{CH4}, Vm_{CO}, kla_{CH4} is calculated with equation S7.
6. Known kla_{CH4}, H_{CH4}, $C_{CH4,in}$ and $C_{L,CH4}$, CH_4-*EC* is calculated with equation S5.
7. Known Q_{in}, $Y_{CH4,in}$, Q_{out}, $Y_{CH4,out}$ and CH_4-*EC*, V_r is calculated with equation S8.
8. Known *V*, Q_{biogas} and Q_{air}, *EBRT* is calculated with equation S9.
9. Known *H/D* and *V*, H_t can be calculated assuming a cylindrical bioreactor.
10. Known *D* and H_t, V_r can be calculated with equation S13.

2. Pumps

All pumps were designed assuming an incompressible behavior of liquids according to the following equation:

$$P = \frac{Q \cdot \Delta P}{\eta} \tag{S14}$$

Where:

P = Power consumption.

Q = Inlet volumetric liquid flow.

ΔP = Pressure difference.

η = Pump electric efficiency.

3. <u>Compressors and blowers</u>

All compressors and blowers were calculated assuming a compressible behavior according to the following equations:

$$P = \frac{P_{is}}{\eta} \tag{S15}$$

$$P_{is} = 2.31 \cdot \frac{\gamma}{\gamma - 1} \cdot \frac{\Delta T}{Pm} \cdot Q \tag{S16}$$

$$\Delta \mathrm{T} = \mathrm{T_{out}} - T_{in} \tag{S17}$$

$$\frac{T_{out}}{T_{in}} = \left(\frac{P_{out}}{P_{in}}\right)^{\frac{\gamma - 1}{\gamma}} \tag{S18}$$

Where:

P =Power consumption.

P_{is} = Isentropic power.

η = Compressor efficiency.

γ = Adiabatic coefficient.

ΔT = Temperature difference.

Pm = Gas molecular weight.

Q = Inlet volumetric gas flow.

T_{out} = Isentropic outlet temperature.

T_{in} = Inlet temperature.

P_{out} = Outlet pressure.

P_{in} = Inlet pressure.

4. Continuous Stirred Tank Reactor (CSTR)

All CSTR have been designed and calculated according to the following equations:

$$HRT = \frac{V}{Q} \quad \text{(S19)}$$

$$V = \frac{\pi \cdot D^2}{4} \cdot H \quad \text{(S20)}$$

$$\frac{H}{D} = 1 \quad \text{(S21)}$$

$$H_t = 1.2 \cdot H \quad \text{(S22)}$$

$$V_r = \frac{\pi \cdot D^2}{4} \cdot H_t \quad \text{(S23)}$$

Where:

HRT = Liquid hydraulic retention time.

V = Liquid volume in the CSTR.

Q = Volumetric inlet liquid flow.

H = Liquid height in the CSTR.

D = Diameter of the CSTR.

H_t = Total height of the CSTR, including overhead.

V_r = Total reactor volume.

5. Ultrafiltration membrane

The ultrafiltration membranes have been designed and calculated according to the following equations:

$$Re_{water} = \frac{Q_P}{Q_{in}} \tag{S24}$$

$$Re_{biomass} = \frac{Q_R \cdot X_R}{Q_{in} \cdot X_{in}} \tag{S25}$$

$$A = \frac{Q_{in}}{P_{flux}} \tag{S26}$$

Where:

Re_{water} = Water recovery.

Q_P = Permeate volumetric flow.

Q_{in} = Inlet volumetric liquid flow.

$Re_{biomass}$ = Biomass recovery.

Q_R = Retentate volumetric flow.

X_R = Biomass concentration in the retentate.

X_{in} = Biomass concentration in the inlet stream.

A = Membrane surface.

P_{flux} = Permeate flux.

6. Electrodialysis

The electrodialysis modules were designed and calculated using the following equations:

$$A = \frac{Q_{in}}{P_{flux}} \tag{S27}$$

$$Re_{salt} = 1 - \frac{1}{FC} \tag{S28}$$

$$Re_{salt} = \frac{Q_R \cdot S_R}{Q_{in} \cdot S_{in}} \tag{S29}$$

$$Re_{water} = \frac{Q_P}{Q_{in}} \tag{S30}$$

Where:

A = Electrodialysis module area.

Q_{in} = Inlet volumetric liquid flow.

P_{flux} = Permeate flux.

Re_{salt} = Salt recovery.

Fc = Salt concentration factor.

Q_R = Retentate volumetric flow.

S_R = Salt concentration in the retentate.

S_{in} = Salt concentration in the inlet liquid stream.

Re_{water} = Water recovery.

Q_P = Permeate volumetric flow.

7. Tray dryer

The tray dryer was calculated using the following equations:

$$A = \frac{V_p}{h} \quad \text{(S31)}$$

$$V_p = \frac{Q_p}{\rho_p} \quad \text{(S32)}$$

$$Re_w = \frac{Q_p \cdot w_p}{Q_{in} \cdot w_{in}} \quad \text{(S33)}$$

$$Q_{air} \cdot k_s = Q_{in} \cdot w_{in} \cdot Re_w \quad \text{(S34)}$$

Where:

A = Surface of tray dryer.

V_p = Dried product volume.

h = Product layer height in the tray dryer.

Q_p = Product volumetric flow.

ρ_p = Product density.

Re_w = Drying efficiency.

W_p = Water content in the product stream.

Q_{in} = Inlet stream volumetric flow.

W_{in} = Water content in the inlet liquid stream.

Q_{air} = Drying air volumetric flow.

K_s = Water solubility in air.

8. Biotrickling filter

The desulfurization biotrickling filter was calculated using the following equations:

$$H_2S - RE = 1 - \frac{C_{out} \cdot Q_{out}}{C_{in} \cdot Q_{out}} \tag{S35}$$

$$H_2S - EC = \frac{C_{in} \cdot Q_{in} - C_{out} \cdot Q_{out}}{V_b} \tag{S36}$$

$$EBRT = \frac{V_b}{Q_{in}} \tag{S37}$$

$$Vb = \frac{\pi \cdot D^2}{4} \cdot H_b \tag{S38}$$

$$\frac{H}{D} = 2 \tag{S39}$$

$$H_t = 1.2 \cdot H_b \tag{S40}$$

$$V_r = \frac{\pi \cdot D^2}{4} \cdot H_t \tag{S41}$$

Where:

$H_2S\text{-}RE$ = Hydrogen sulfide removal efficiency.

C_{out} = Hydrogen sulfide concentration in the outlet stream.

Q_{out} = Outlet gas volumetric flow.

C_{in} = Hydrogen sulfide concentration in the inlet stream.

Q_{in} = Inlet gas volumetric flow.

$H_2S\text{-}EC$ = Hydrogen sulfide elimination capacity.

V_b = Packed bed volume.

$EBRT$ = Empty bed residence time.

D = Biotrickling filter diameter.

H_b = Packed bed height.

H_t = Total biotrickling filter height, including overhead.

V_r = Biotrickling filter volume.

9. Ionic exchange column

The ionic exchange columns were calculated according to the following equations:

$$Re_{ectoine} = \frac{m_{ectoine_{adsorbed}}}{Q{\cdot}C_{in_{ectoine}}} \tag{S42}$$

$$Q_{resin} = \frac{m_{ectoine_{adsorbed}}}{m_{resin}} \tag{S43}$$

$$\rho_{resin} = \frac{m_{resin}}{V_{resin}} \tag{S44}$$

$$BV = \frac{Q{\cdot}C_{in_{ectoine}}{\cdot}Re_{ectoine}}{Q_{resin}{\cdot}\rho_{resin}} \tag{S45}$$

$$BV = \frac{\pi{\cdot}D^2}{4}{\cdot}H_c \tag{S46}$$

$$\frac{H_c}{D} = 10 \tag{S47}$$

Where:

$Re_{ectoine}$ = Ectoine recovery efficiency.

$m_{ectoine\ adsorbed}$ = Mass of ectoine adsorbed in the ionic exchange resin.

Q = Inlet volumetric liquid flow.

$C_{in,ectoine}$ = Ectoine concentration in the inlet liquid stream.

Q_{resin} = Resin adsorption capacity.

m_{resin} = Mass of ionic exchange resin in the packed bed.

ρ_{resin} = Resin density.

V_{resin} = Resin volume.

BV = Bed volume in the packed bed column.

D = Column diameter.

H_c = Bed height.

10. Spray dryer

The spray dryer has been calculated according to the following equation:

$$V = Ev_{rate} \cdot Q \tag{S48}$$

Where:

V = Volume of the spray drying unit.

Ev_{rate} = Specific evaporation rate.

Q = Inlet volumetric liquid flow.

11. Centrifuge

All centrifuges have been calculated according to the following equations:

$$Re_{water} = \frac{Q_R}{Q_{in}} \tag{S49}$$

$$Re_{solid} = \frac{Q_R \cdot C_R}{Q_{in} \cdot C_{in}} \tag{S50}$$

$$C_f = \frac{C_R}{C_{in}} \tag{S51}$$

Where:

Re_{Water} = Water recovery efficiency.

Q_R = Retentate volumetric flow.

Q_{in} = Inlet volumetric liquid flow.

Re_{Solid} = Solid (biomass or ectoine) recovery efficiency.

C_R = Solid (biomass or ectoine) concentration in the retentate stream.

C_{in} = Solid (biomass or ectoine) concentration in the inlet stream.

C_f= Solid (biomasss or ectoine) concentration factor.
